# Blockade of the ADAM8-Fra-1 complex attenuates neuroinflammation by suppressing the Map3k4/MAPKs axis after spinal cord injury

**DOI:** 10.1186/s11658-024-00589-3

**Published:** 2024-05-16

**Authors:** Zhanyang Qian, Rulin Li, Tianyu Zhao, Kunxin Xie, PengFei Li, Guangshen Li, Na Shen, Jiamin Gong, Xin Hong, Lei Yang, Haijun Li

**Affiliations:** 1 Department of Orthopedics, Taizhou People’s Hospital of Nanjing Medical University, Taizhou School of Clinical Medicine, Nanjing Medical University, Taizhou, China; 2 Department of Orthopedics, Zhongda Hospital of Southeast University, Nanjing, China; 3 School of Postgraduate, Dalian Medical University, Dalian, China; 4 Key Laboratory of Human Functional Genomics of Jiangsu Province, Department of Biochemistry and Molecular Biology, Nanjing Medical University, Nanjing, China; 5 School of Postgraduate, Nanjing University of Chinese Medicine, Nanjing, China; 6 School of Basic Medicine, Nanjing Medical University, Nanjing, China

**Keywords:** ADAM8, Fra-1, Spinal cord injury, Microglia, Neuroinflammation, Map3k4

## Abstract

**Background:**

Mechanical spinal cord injury (SCI) is a deteriorative neurological disorder, causing secondary neuroinflammation and neuropathy. ADAM8 is thought to be an extracellular metalloproteinase, which regulates proteolysis and cell adherence, but whether its intracellular region is involved in regulating neuroinflammation in microglia after SCI is unclear.

**Methods:**

Using animal tissue RNA-Seq and clinical blood sample examinations, we found that a specific up-regulation of ADAM8 in microglia was associated with inflammation after SCI. In vitro*,* microglia stimulated by HMGB1, the tail region of ADAM8, promoted microglial inflammation, migration and proliferation by directly interacting with ERKs and Fra-1 to promote activation, then further activated Map3k4/JNKs/p38. Using SCI mice, we used BK-1361, a specific inhibitor of ADAM8, to treat these mice.

**Results:**

The results showed that administration of BK-1361 attenuated the level of neuroinflammation and reduced microglial activation and recruitment by inhibiting the ADAM8/Fra-1 axis. Furthermore, treatment with BK-1361 alleviated glial scar formation, and also preserved myelin and axonal structures. The locomotor recovery of SCI mice treated with BK-1361 was therefore better than those without treatment.

**Conclusions:**

Taken together, the results showed that ADAM8 was a critical molecule, which positively regulated neuroinflammatory development and secondary pathogenesis by promoting microglial activation and migration. Mechanically, ADAM8 formed a complex with ERK and Fra-1 to further activate the Map3k4/JNK/p38 axis in microglia. Inhibition of ADAM8 by treatment with BK-1361 decreased the levels of neuroinflammation, glial formation, and neurohistological loss, leading to favorable improvement in locomotor functional recovery in SCI mice.

**Graphical Abstract:**

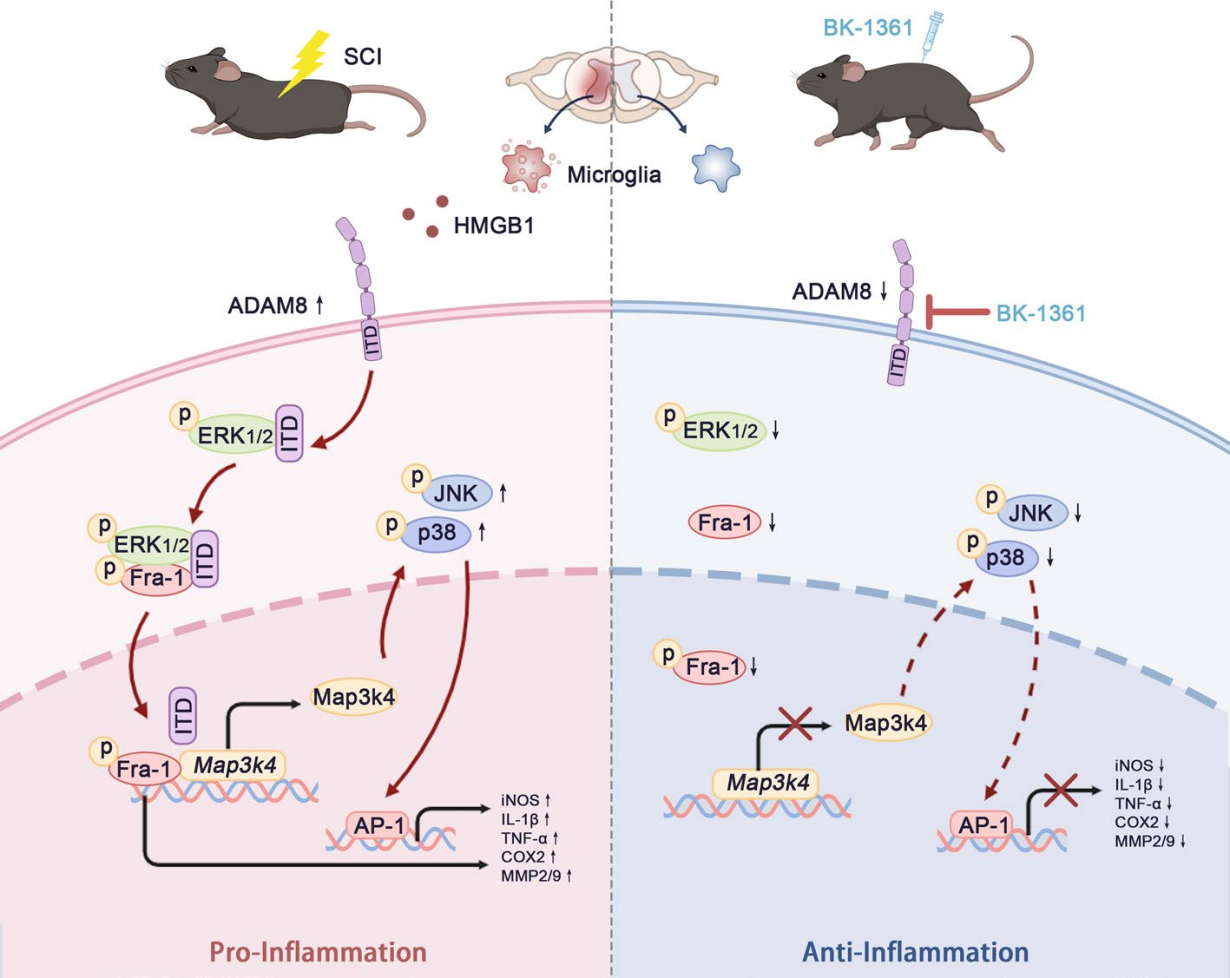

**Supplementary Information:**

The online version contains supplementary material available at 10.1186/s11658-024-00589-3.

## Introduction

Mechanical spinal cord injury (SCI) is a deteriorative neurological disorder resulting from violent traumas such as falls, vehicle accidents, and bullet wounds [1–3]. The primary damages cause immediate neuro-histological destruction and neural cell death, which further releases various damage-associated molecular patterns (DAMPs) molecules, triggering excessive neuroinflammation [4, 5]. The secondary neuroinflammation process shifts microglia from a resting state to an activated mode [6], and the inflammation-activated microglia rapidly proliferate and migrate to injured foci of spinal cords [7], where they secrete multiple molecular proteins including cytokines, chemokines, and proteases [8–10]. Previous studies have reported that the inflammatory activities of microglia after SCI induced the development of neuropathies and subsequent locomotor dysfunctions [11–13], but the molecular mechanism involved in this microglia-induced neuroinflammation is currently unknown.

High mobility group box-1 protein (HMGB1), originally thought to be a highly conserved protein, is a DNA-binding protein involved in stabilization of gene expressions and nucleosomes [14]. Increasing studies have reported that HMGB1 derived from immune cells or cells undergoing cell death functioned as a crucial mediator of inflammation [15, 16]. It was found that an increase in HMGB1 expression by damaged neurons and activated microglia promoted neuroinflammation after SCI [17, 18]. Known as a classical DAMP, HMGB1 binds with the Toll-like receptor 4 and the receptor for advanced glycation end products, to activate the downstream NF-κB and MAPK signaling axis involved in the transcriptional promotion of inflammation-related genes in microglia [19, 20]. Previous studies reported that inhibiting HMGB1-induced inflammatory pathways protected against the pathological processes of mechanical SCI, as well as that of spinal cord ischemia–reperfusion injury [21–23]. However, HMGB1 also induces neural regeneration by promoting axonal growth and neural differentiation of stem cells [18, 24]. Therefore, for a better recovery after SCI, it is important to inhibit the signal axis of HMGB1 targeting microglia.

A disintegrin and a metalloproteinase domain 8 (ADAM8) are members of a transmembrane and secretory protein family, which regulate proteolysis and cell adherence on the cell surface [25, 26]. ADAM8 protein consists of seven regions, including a pro-domain, metalloproteinase domain, disintegrin domain, cysteine-rich domain, EGF-like domain, transmembrane domain, and a cytoplasmic tail [26, 27]. ADAM8 usually increases and induces catalysis, degradation, adhesion, and other functions during multiple pathologies, while the cytoplasmic tail of ADAM8 is involved in the regulation of intracellular signal transduction [28–30]. Increasing studies [31–33] have reported that ADAM8 played a vital role in airway inflammation in diseases of the respiratory system, such as acute respiratory distress syndrome (ARDS), asthma, and chronic obstructive pulmonary disease (COPD). However, the role and molecular mechanism of ADAM8 in SCI remains unclear.

In the current study, we identified the function of ADAM8 in microglia during HMGB1-induced neuroinflammation after SCI. We found that ADAM8 was a crucial early biomarker in the serum of SCI patients, and that it was positively correlated with inflammation. In microglia after HMGB1 insult, ADAM8 regulated the levels of neuroinflammation, proliferation, and migration by interacting with Fra-1 to stabilize its phosphorylation, which further activated MAPK signaling by promoting Map3k4 transcription. BK-1361, a selective inhibitor of ADAM8, was used to treat SCI mice, resulting in significant decreases of secondary neuropathies and increases in the recoveries of locomotor functions during post-SCI.

## Material and methods

### Blood specimens of SCI patients

We collected blood specimens (Table S1) from 12 SCI patients within 3 days after injury, and from another 12 admitted patients, who will undergo percutaneous pedicle screw removal as controls. All patients were admitted to the Department of Orthopedics at the Taizhou People’s Hospital of Nanjing Medical University. The collected serum and blood cells were separated by centrifugation at 1300 rpm, 4 °C for 5 min, then stored at – 80 ℃ until further studied.

### Isolation and culture of primary microglia

A 75 cm^2^ culture flask was coated by Poly-Lysine (Gibco, Grand Island, NY, USA) for 1 h, washed with phosphate-buffered saline (PBS) three times, then incubated at 37 °C overnight. The next day, neonatal 3-day-old C57BL/6 mice were euthanized and immersed in 75% ethanol for 5 min. The brains of the mice were placed in PBS containing 2% fetal bovine serum (FBS; Gibco) and the micro-vessels and the pia mater on the brains were removed using a stereomicroscope. We then extracted and collected the cortex in Dulbecco’s Modified Eagle Medium (DMEM, KeyGEN, Nanjing, China) and dissected them into 2 × 2 mm pieces on ice. After centrifugation at 800 rpm at 4 °C for 3 min, the sedimentary pieces were resuspended in 0.25% trypsin (Gibco) for a 5 min digestion at 37 °C. We then added FBS to terminate the digestion, followed by a cell filtration using a 200-mesh cell screen. The filtered solution was centrifuged at 1000 rpm for 5 min, then the cells were resuspended in DMEM containing 10% FBS and 1% Pen/Strep (KeyGEN) and seeded into polylysine-coated culture flasks at a density of 10^7^ cells/mL for culturing. We changed the medium once every 72 h. After culturing for 2–3 weeks, mature microglia were sedimented using 200 rpm for 4 h, and collected for further studies. For stimulation of neuroinflammation, the microglia were treated with mouse HMGB1 protein (100 ng/mL; MedChemExpress, Shanghai, China) for specific times as indicated.

### Animals and grouping

All the animal experiments were conducted in accordance with the National Institutes of Health guidelines on the care and use of laboratory animals, and were approved by the Animal Care Committee of Nanjing Medical University. Adult male C57BL/6J mice (8-weeks-old, with an average weight of 20 g) were obtained from HuaChuang Sino (Taizhou, Jiangsu, China). The mice had ad libitum access to water and food, were kept at 22 ± 2 °C, 60 ± 5% humidity, and a 12 h-cycle of light/dark. Mice were randomly divided into four groups: (1) the Saline group, in which the mice were treated with a T10 laminectomy and injected with saline; (2) the SCI + Saline group, in which the mice were induced with SCI and injected with saline; (3) the SCI + 10 mg/kg BK-1361 group, in which the SCI mice were treated with 10 mg/kg BK-1361; and (4) the SCI + 25 mg/kg BK-1361 group, in which the SCI mice were treated with 25 mg/kg BK-1361.

### SCI modeling

In accordance with our previous study [34], we anesthetized the mice by intraperitoneal injection of sodium phenobarbitol, sterilized their skin using iodophor, and then exposed the lamina at T10. Their spinal cords were impacted using a 5 g of a spinal cord impactor (RWD, Shenzhen, China) falling from a height of 5 cm. The SCI modeling was successful when the mice presented a hematoma in their spinal cord along with a tail-flick and paralysis of hind limbs. We intraperitoneally administered the ADAM8 inhibitor, BK-1361 (Macklin, Shanghai, China), dissolved in saline, to the SCI mice for 7 days after SCI, while the remaining groups were injected intraperitoneally with an equivalent amount of PBS. The first administration of BK-1361 was at 6 h after SCI modeling. The SCI mice were assisted daily during urination until their natural urination reflex returned.

### RNA-seq analysis

The mice were sacrificed at 1, 3, and 7 days after SCI, and their spinal cords were removed for RNA extraction using TRIzol reagent (YiFeiXue Biotechnology, Nanjing, China). RNA was sequenced from RNA-Seq libraries using the Illumina HiSeq 4000 sequencer (Biomarker Technologies, Beijing, China) and gene expressions were quantified by Tophat and Cufflinks. Using the Gene Ontology (GO) and Kyoto Encyclopedia of Genes and Genomes (KEGG) databases, differences of gene expressions in biological enrichments and gene pathways were analyzed and annotated. Gene heat maps were then used to show expression differences of genes. Genes with fold-change ≥ 2 and *P*-value < 0.01 were defined as differentially expressed genes.

### Public single cell data processing

The public single cell data used in this study from a previously published study [35], and the downloaded data was stored with the Figshare (10.6084/m9.figshare.17702045). We obtained single cell RDS files from the website provided by the previous authors, and made cell annotations consistent with the original text. The cleaning, dimensionalization, clustering, and cell annotation of single cell data are all completed by the Seurat package.

### Lentivirus (LV) and plasmid transfection

The microglia were transfected for ADAM8 mRNA interference (ADAM8i) using lentivirus loaded with shRNA of ADAM8 in DMEM, supplemented 10% FBS and 1 × HitransG P (GeneChem, Shanghai, China) for 12 h, when the cell confluence was ~ 40%. Then, the cells were further cultured in DMEM supplemented with 10% FBS for 60 h. The HEK293T cells obtained from Procell Life Technology (No. CL-0005, Wuhan, China) were seeded in DMEM supplemented 10% FBS and 1% Pen/Strep. Total of 2.5 μg plasmids containing full-length or deleted Flag-ADAM8 (GeneChem) were co-transfected with a plasmid containing His-Fra-1(GeneChem). After incubation for 48 h at 37 °C, the cells were harvested for the co-immunoprecipitation (Co-IP) assay.

### Co-IP assay

We harvested the cells using cell lysis buffer [(Beyotime, Shanghai, China) supplemented with 1 mM phenylmethanesulfonyl fluoride (KeyGEN)] for western blotting (WB) and IP. After centrifugation at 12,000×*g*, at 4 ℃ for 10 min, 50 μL supernatant was removed and probed with positive antibodies or IgG antibody using Protein G Sepharose™ 4 Fast Flow beads (GE Healthcare, Stockholm, Sweden) overnight at 4 °C. The beads were then washed five times for 5 min with centrifugation at 500×*g* and 4 ℃, after each wash, then the beads were resuspended in 2 × loading buffer and boiled at 100 °C.

### Real-time quantitative reverse transcription PCR (qRT-PCR)

Human blood cells or murine injured spinal cords were lysed on ice in TRIzol reagent (YiFeiXue Biotechnology) to extract RNA. We then purified the RNA using trichloromethane and isopropanol and measured the concentration at 260/280 nm using a Nano 2000 (vendor and location). Synthesis of cDNA was performed using a Yfx 1st Strand cDNA Synthesis Kit (Vazyme, Nanjing, China) and qRT-PCR analysis was carried out using a RT SuperMix qPCR Kit (Vazyme) using a Roche LightCycler 480 (Roche, Basel, Switzerland). We determined the relative expression of genes using the ^ΔΔ^CT method, with the expression of β-actin used for normalization. The primer sequences used in the present study are listed in Table 1.

**Table 1:** The primers of qRT-PCR in the study

Gene name	Forward sequence (5′–3′)	Reverse sequence (5′–3′)
ADAM8	TGCTCAGCGTCTTATGGACAC	AGGCCAAACCACTTCATACTG
GAPDH	AGGTCGGTGTGAACGGATTTG	GGGGTCGTTGATGGCAACA
Fosl-1	ATGTACCGAGACTACGGGGAA	CTGCTGCTGTCGATGCTTG
Map3k	AGGCAGGAGTGCATGTTGG	AAGTCCTCTGGATCGGATTCC

### Western blots (WB)

Total protein from microglia or the injured spinal cord was extracted using a Total Protein Extraction Kit (KeyGEN) according to the manufacturer’s protocol. The concentration of protein was quantified using the bicinchoninic acid method. Equivalent protein was resolved using a PAGE Gel Fast Preparation Kit (Epizyme, Shanghai, China) for electrophoresis, then transferred to a polyvinylidene fluoride (PVDF) membrane (Millipore, Boston, MA, USA), and blocked using 5% skim milk (BioFroxx, Guangzhou, China) dissolved in Tris-buffered saline-Tween 20 (Biosharp, Hefei, China) at room temperature. The PVDF membrane was then rinsed and probed with primary antibodies overnight at 4 °C, and further incubated at room temperature with secondary antibodies labeled with horse radish peroxidase. The details of these antibodies are listed in Table 2. The protein signals were captured using an automatic chemiluminescence image analysis system (Tanon, Shanghai, China) and quantified using ImageJ software (National Institutes of Health, Bethesda, MD, USA).

**Table 2:** The information of antibodies we used in the study

Antibodies name #Cat. No.	Source	Species	Application	Dilution rate
Anti-iNOS antibody #ab15323	Abcam	Rb	WB	1:250
Anti- MS2 Rabbit mAb #R25016	ZEN-BIOSCIENCE	Rb	WB	1:1000
Anti-Fra-1 antibody (D-3) #sc-376148	Santa Cruz Biotechnology	Ms	WB	1:1000
Anti-MMP2 antibody (8B4) #sc-13595	Santa Cruz Biotechnology	Ms	WB	1:500
Anti-MMP9 antibody (E-11) #sc-393859	Santa Cruz Biotechnology	Ms	WB	1:500
Anti-ZO-1 antibody (R40.76) #sc-33725	Santa Cruz Biotechnology	Rat	WB	1:1000
Anti-Occludin antibody (E-5) #sc133256	Santa Cruz Biotechnology	Ms	WB	1:1000
Cox2 (D5H5) XP^®^ Rabbit mAb #12282	CST	Rb	WB	1:1000
Anti-Phospho-p44/42 MAPK (Erk1/2) (Thr202/Tyr204)(D13.14.4E)XP^®^ Rabbit mAb #4370	CST	Rb	WB	1:1000
Anti-p44/42 MAPK (Erk1/2) (137F5) Rabbit mAb #4695	CST	Rb	WB	1:1000
Anti-P38 MAPK Monoclonal antibody #66234-1-Ig	Proteintech	Ms	WB	1:1000
Anti-Phospho-P38 MAPK (Thr180/Tyr182) Polyclonal antibody #28796-1-AP	Proteintech	Ms	WB	1:1000
Anti-Phospho-SAPK/JNK (Thr183/Tyr185) (81E11) Rabbit mAb #4668	CST	Rb	WB	1:1000
Anti-JNK1 + JNK2 + JNK3(phospho T183 + T183 + T221) (EPR5693) Rb #ab124956	Abcam	Rb	WB	1:1000
Anti-Phospho-FRA1 (Ser265) Antibody Rabbit #3880	CST	Rb	WB	1:1000
Anti-Histone H3 Monoclonal antibody #68345-1-Ig	Proteintech	Ms	WB	1:5000
6*His, His-Tag Monoclonal antibody #66005-1-Ig	Proteintech	Ms	WB	1:5000
DYKDDDDK tag Monoclonal antibody (Binds to FLAG^®^ tag epitope) #6008-4-Ig	Proteintech	Ms	WB	1:5000
HRP-conjugated GAPDH Monoclonal antibody # HRP-60004	Proteintech	Ms	WB	1:10,000
HRP-conjugated Beta Actin Monoclonal antibody #HRP-60008	Proteintech	Ms	WB	1:10,000
HRP-conjugated Alpha Tubulin Monoclonal antibody #HRP-66031	Proteintech	Ms	WB	1:10,000
Goat Anti-Rabbit IgG Secondary antibody (H + L), HRP #YFSA02	YIFEIXUE BioTech	Goat	WB	1:10,000
Goat Anti-Mouse IgG Secondary antibody (H + L), HRP #YFSA01	YIFEIXUE BioTech	Goat	WB	1:10,000
Rabbit Anti-Rat IgG Secondary antibody (H + L), HRP #YFSA04	YIFEIXUE BioTech	Rb	WB	1:10,000
Anti-MS2 Rabbit mAb #R25016	ZEN-BIOSCIENCE	Rb	IP	1:20
Anti-Phospho-p44/42 MAPK (Erk1/2) (Thr202/Tyr204)(D13.14.4E)XP^®^ Rabbit mAb #4370	CST	Rb	IP	1:50
Anti-Phospho-FRA1 (Ser265) Antibody Rabbit #3880	CST	Rb	IP	1:50
Anti-Phospho-SAPK/JNK (Thr183/Tyr185) (81E11) Rabbit mAb #4668	CST	Rb	IP	1:50
Mouse IgG (Sepharose^®^ Bead Conjugate) #3420	CST	Ms	IP	1:20
Anti-Fra-1 antibody (D-3) #sc-376148	Santa Cruz Biotechnology	Ms	ChIP	1:100
Anti-MS2 antibody #ab236949	Abcam	Rb	IF	1:200
Anti-Fra-1 antibody (D-3) #sc-376148	Santa Cruz Biotechnology	Ms	IF	1:100
Anti-iNOS antibody (ab15323)	Abcam	Rb	IF	1:100
Anti-Iba1 antibody [EPR16588] #ab178846	Abcam	Rb	IF	1:500
Cox2 (D5H5) XP^®^ Rabbit mAb #12282	CST	Rb	IF	1:500
Anti-MMP2 antibody (8B4) #sc-13595	Santa Cruz Biotechnology	Ms	IF	1:100
Anti-MMP9 antibody (E-11) #sc-393859	Santa Cruz Biotechnology	Ms	IF	1:100
Rat Anti-Mouse CD31 antibody #550274	BD Biosciences	Ms	IF	1:100
GFAP (GA5) Mouse mAb #3670	CST	Ms	IF	1:600
Neurofilament-H (RMdO 20) Mouse mAb #2836	CST	Ms	IF	1:400
Anti-NeuN antibody #MAB377	Merck Millipore	Ms	IF	1:100
Anti Myelin basic protein antibody # ab313827	Abcam	Rb	IF	1:400
Alexa Fluor^®^ 594 AffiniPure Fab Fragment Goat Anti-Rabbit IgG (H + L) #111587003	Jackson ImmunoResearch	Goat	IF	1:500
Alexa Fluor^®^ 488 AffiniPure Fab Fragment Goat Anti-Rabbit IgG (H + L) #111547003	Jackson ImmunoResearch	Goat	IF	1:500
Alexa Fluor^®^ 594 AffiniPure F(ab′)_2_ Fragment Goat Anti-Mouse IgG (H + L) #115586003	Jackson ImmunoResearch	Goat	IF	1:500
Alexa Fluor^®^ 488 AffiniPure F(ab′)_2_ Fragment Goat Anti-Mouse IgG (H + L) #115546003	Jackson ImmunoResearch	Goat	IF	1:500

### Chromatin immunoprecipitation sequencing (ChIP-Seq)

A BV2 microglia cell line (No. CL-0493A) was obtained from the Procell Life Technology and cultured with DMEM with 10% FBS and 1% Pen/Strep, then treated with 100 ng/mL HMGB1 for 9 h. Then, 2 × 10^7^ cells were crosslinked with 1% formaldehyde for 10 min at room temperature, followed by termination of the reaction using 0.125 M glycine. The cells were harvested in ChIP lysis buffer and the nuclei were extracted by centrifugation at 2000 × *g* for 5 min. The nuclei were resuspended in nucleus lysis buffer and sonicated to a length of 200–500 bp DNA. We then stored 10% volume as “input”, and Fra-1 antibody was added to 80% of the volume for ChIP at 4 °C, while the remaining 10% was incubated with IgG antibody. After DNA was extracted using the phenol–chloroform method, the DNA was measured using a NovaSeq 6000 sequencer with a PE150 model system (Illumina, San Diego, CA, USA). The sequencing data were filtered using Trimmomatic (version 0.36). MACS2 software (version 2.1.1) was used for peak identification with a corrected *P*-value cutoff of 0.05. The de novo motif of the Fra-1 binding site was analyzed using HOMER software. Statistically significant enrichments for annotated genes were conducted by GO and KEGG analyses using KOBAS software (version 2.1.1).

### Transwell assay

The microglia after specific treatment in each group were resuspended in 200 µL of FBS-free DMEM and seeded on the upper layer of inserts; 500 µL of DMEM containing 10% FBS was then added to each well of a 24-well plate. The cells were then cultured for 48 h and fixed with 4% paraformaldehyde (PFA; Servicebio, Wuhan, China) for 15 min. We stained the microglia using a Crystal Violet Staining Kit (KeyGEN) for 20 min, following the manufacturer’s instructions. The cells on the upper layer were washed with water, then dabbed with cotton swabs, while the migrated cells on the lower surface were counted using a microscope (Nikon, Tokyo, Japan).

### Cell proliferation assay

We assessed cell proliferation using a kFlour488-EdU Cell Proliferation Detection Kit (KeyGen). Briefly, the 2 × 5-bromo-2-deoxyuracil (EdU) working buffer was premixed with equivalent medium volume, then the cells were probed with the mixture for 2 h at 37 °C. After a fixation using 4% PFA for 20 min, microglia were treated with 0.5% Triton X-100 (Biosharp, Hefei, China) for 20 min, and then incubated with the Click-iT reaction solution for 30 min in the dark. Finally, images were captured using a fluorescence microscope (Leica, Wetzlar, Germany).

### Immunofluorescence (IF) staining

The mice were sacrificed and perfused with PBS and 4% PFA by intracardiac injection under anesthesia. We removed their spinal cords and immersed them in 4% PFA for 24 h. The tissues then were embedded in paraffin and cut into 5-μm sections. After the sections were deparaffinized, hydrated, antigen repaired, and immuno-blocked, IF staining was conducted. The cells were fixed with 4% PFA for 15 min before IF staining. The sections or cells were incubated overnight with the primary antibodies, and then with the fluorescent secondary antibodies for 1 h in the dark. The antibodies of interest are listed in Table 2. The nucleus was counterstained using a DAPI Staining Reagent (Servicebio). We captured fluorescence images using a fluorescent microscope (Leica) and analyzed the fluorescence intensities using the ImageJ software.

### ELISAs

The cells were lysed in Protein Lysis Buffer (KeyGEN) and mixed using an electronic oscillator. After centrifugation at 12,000 rpm and 4 °C for 15 min, the supernatant was collected and stored at − 80 °C. According to the manufacturer’s protocol, the levels of IL-1β and TNF-α in the supernatant were measured using mouse IL-1β and TNF-α ELISA Kits, respectively (Lianke Bio, Hangzhou, China). The level of ADAM8 in patient serum was determined using a Human ADAM8 ELISA Kit (Enzyme-linked Bio, Shanghai, China). We measured the absorbance at 450 nm using a microplate reader (BioTek, Winooski, VT, USA).

### Histological staining

- Hematoxylin–eosin (HE): The sections were stained with HE Staining Reagent (Servicebio) according to the manufacturer’s instructions. We dehydrated the sections in graded ethanol and vitrificated them in dimethylbenzene.
- Nissl Staining: We performed Nissl staining of the tissues using Nissl Staining Reagent (Servicebio), according to the manufacturer’s guidelines. After deparaffination and hydration, the sections were stained the Nissl body stain, using 0.5% toluidine blue for 5 min. The sections then were differentiated with 0.1% glacial acetic acid and sealed with neutral balsam.
- Luxol Fast Blue (LFB) staining: To visualize the morphological structure and pathological changes of the myelin sheath post-SCI, LFB staining was performed using the LFB Staining Reagent (Servicebio). The sections were stained with the working buffer for 40 min and dehydrated using absolute ethanol three times for 5 min each, then vitrificated using dimethylbenzene for 5 min and mounted with neutral balsam.

All photographs were captured using a microscope (Nikon).

### Behavioral assessment

The function of hind limbs was evaluated using the Basso Mouse Scale (BMS) [35] and the walking track assay [36]. Two investigators observed the motion of the mouse ankle joint, the plantar and dorsal contact with the ground, the stability of the trunk, and the position of the mouse tail when they were allowed to crawl freely in an open field for 4 min using a double-blind method. The average value of the hind limb scores was recorded.

### Statistical analysis

All data are presented as the mean ± SEM and drawn using Prism software (version 8.3, GraphPad, San Diego, CA, USA). Statistical analysis between two groups was conducted using the unpaired Student’s *t*-test, and analyses of more than two groups used one-way or two-way analysis of variance, followed by Tukey’s post-hoc test. Pearson’s correlation coefficient was used to evaluate all correlations. A value of *P* < 0.05 was defined as statistical significance.

## Results

### High transcription of ADAM8 and Fosl-1 during the acute phase after SCI

**Fig. 1:**
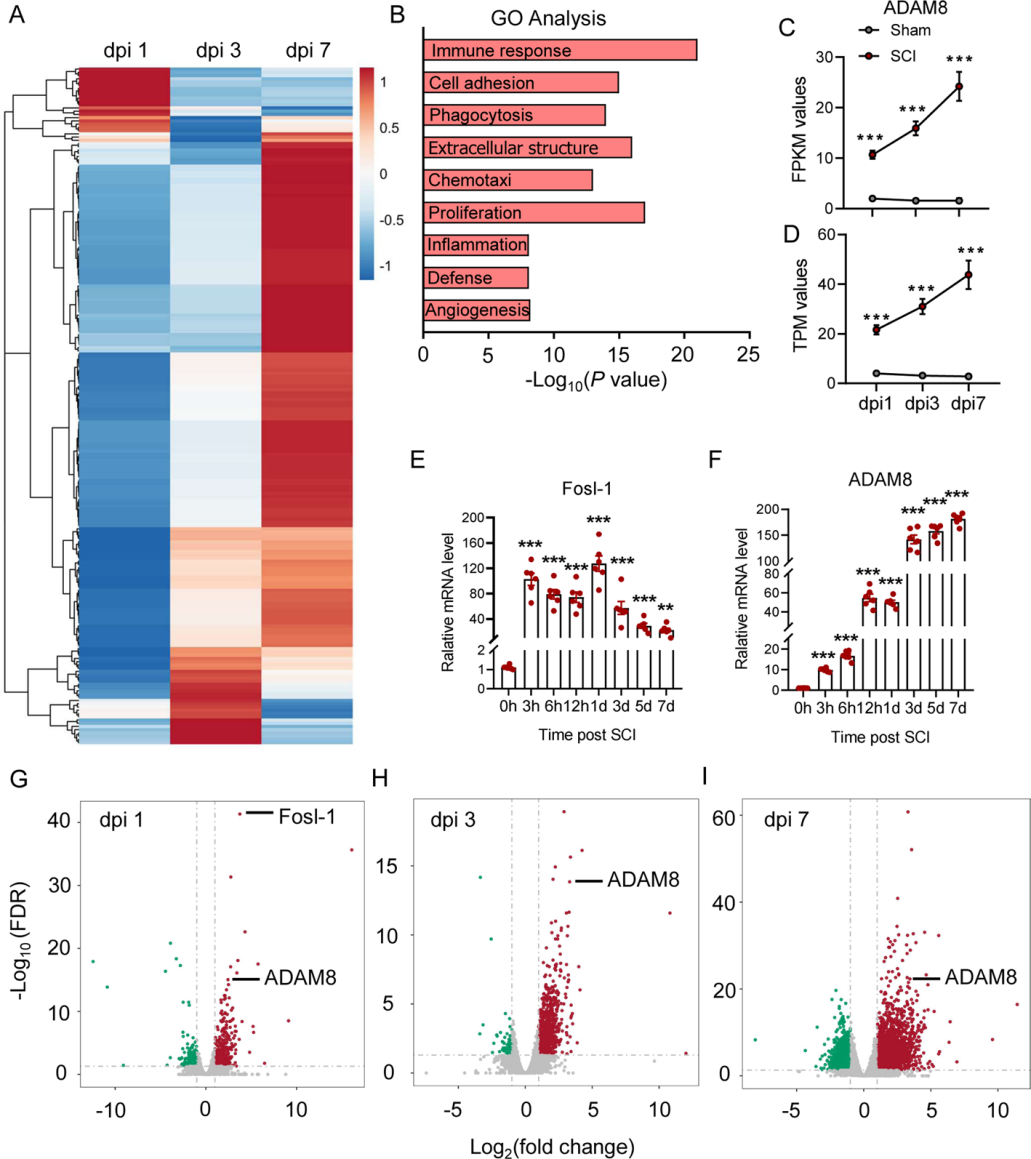
ADAM8 and Fosl-1 transcription increases during the acute phase of SCI.** A** RNA-seq data at dpi 1, 3 and 7 after SCI in mice. **B** The differentially-expressed genes by GO analysis.** C** The fragments per kilobase of million (FPKM) reads of ADAM8 in the SCI mice from dpi 1 to dpi 7 in mice. **D** The transcripts per million (TPM) kilobases of ADAM8 in the SCI mice from dpi 1 to dpi 7 in mice in mice. **E, F** The mRNA levels of Fosl-1 (**E**) and ADAM8 (**F**) increase after SCI in mice. **G** Fosl-1 and ADAM8 were the differential genes expression at dpi 1. **H, I** ADAM8 was the differential genes expression at dpi 3(**H**) and 7(**I**). *p < 0.05 vs. sham group (**C**, **D**), *p < 0.05 vs. 0 h (**E**, **F**) by one-way ANOVA followed by Tukey's post hoc analysis (*p < 0.05, **p < 0.01, and ***p < 0.001)

The analysis of RNA-seq data showed reprogramming of gene expressions during the acute phase after SCI (Fig. 1A). Furthermore, GO analysis suggested that these differentially-expressed genes were involved in immune response, cell adhesion, phagocytosis, extracellular structure, chemotaxis, cell proliferation, inflammation, and angiogenesis (Fig. 1B). ADAM8 and Fra-1 (also named Fosl-1) were two noticeable genes at the acute phase after SCI. It was found that both of the fragments per kilobase of million reads and the transcripts per million kilobases of ADAM8 in the SCI mice gradually increased from dpi 1 to dpi 7 (Fig. 1C, D), suggesting that relative gene expressions of ADAM8 were up-regulated after SCI. Increases in Fosl-1 and ADAM8 were also found at the transcription level after SCI (Fig. 1E, F). Volcano plots showed that Fosl-1 and ADAM8 were the differentially-expressed genes at dpi 1 (Fig. 1G), and ADAM8 showed continued differential expression at the acute phase after SCI (Fig. 1G–I). Therefore, we sought to investigate the role and molecular mechanism of ADAM8 during neuroinflammation after SCI.

### ADAM8 shows a high correlation with neuroinflammation in microglia post SCI

**Fig. 2:**
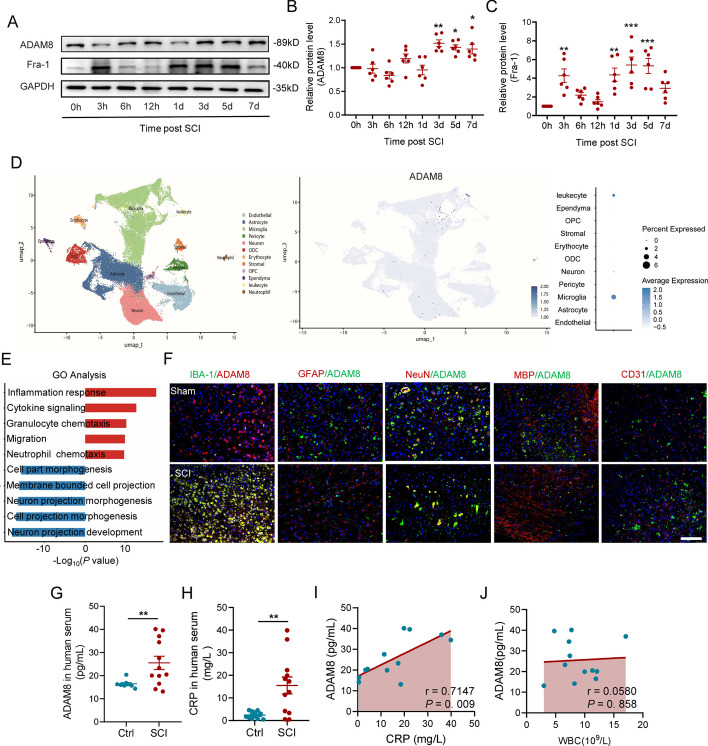
ADAM8 and Fosl-1 is highly associated with microglia after SCI.** A–C** ADAM8 and Fra-1 remarkably increased from 1 to 7 dpi in mice. **D** UMAP visualization plot of 59,558 spinal cord cells sequenced from all samples in the public dataset and their cell types. Feature Plot of the ADAM8’s expression level in UMAP visualization. Dot Plot of the ADAM8’s expression level in the single-cell dataset of different types of cells. **E** Bidirectional bar chart of differential gene enrichment pathways in our RNA-seq data. **F** ADAM8 co-localized with IBA-1 positive microglia/macrophages at 3 dpi after SCI in mice, but not astrocytes, neuronal, oligodendrocytes, and microvascular endothelial cells. Scale bar, 100 μm. **G, H** The levels of ADAM8 (**G**) and CRP (**H**) in patients within 3 days after SCI in human serum specimens, were significantly higher than control patients. **I, J** There is a high correlation between ADAM8 levels and CRP (**I**) levels in SCI patients’ serum specimens, but not with the number of white blood cells (**J**). *p < 0.05 vs. 0 h (**B,** **C**), *p < 0.05 vs. Ctrl group (**G**, **H**) by one-way ANOVA followed by Tukey's post hoc analysis (*p < 0.05, **p < 0.01, and ***p < 0.001)

We next refined the time period after SCI and determined the expressions of ADAM8 protein and Fosl-1-coded protein Fra-1. The expression of ADAM8 showed little changes in protein levels within 1 dpi, but Fra-1 showed an increased peak at 3 h post-SCI, then decreased to the original level at 1 dpi; afterwards, we found that both ADAM8 and Fra-1 remarkably increased from 1 to 7 dpi (Fig. 2A–C). Moreover, we analyzed public single cell data, the uniform manifold approximation and projection for dimension reduction (UMAP) showed that the expression of *ADAM8* gene enriched in microglia and leukocyte after SCI (Fig. 2D). Go analysis of ADAM8 also exhibited a positive correlation to inflammation response, cytokine-mediated signaling, cell chemotaxis and migration (Fig. 2E). The expression location of ADAM8 in various neural cells was then traced using IF staining, which consistently showed that ADAM8 was minimally expressed in GFAP positive astrocytes, and MBP positive oligodendrocytes, as well as CD31 positive microvascular endothelial cells, regardless of SCI; however, we also found that the expression of ADAM8 in IBA-1 positive microglia/macrophages increased but decreased in NeuN positive neurons at 3 days after SCI (Fig. 2F). It was suggested that the increased level of ADAM8 might be involved in microglia-induced immune and inflammation responses after SCI. In human serum specimens, the levels of exocrine ADAM8 in patients within 3 days after SCI were significantly higher than those in control patients (Fig. 2G), along with an increase in the levels of the inflammatory biomarker, C-reaction protein (CRP) (Fig. 2H). Further analysis showed a high correlation (*r* = 0.7147) with statistical significance between the levels of ADAM8 and the CRP in SCI patients (Fig. 2I), suggesting that ADAM8 was a crucial indicator of inflammation, post-SCI. However, it did not exhibit a significant correlation in the levels of ADAM8 and white blood cells (Fig. 2H). Using in vitro HMGB1-treated neuroinflammation in microglia, we found that the protein level of ADAM8 peaked at 9 h post treatment (Fig. S1A and S1B); therefore, a 9 h-treatment of HMGB1 was selected to investigate the role of ADAM8 in microglia during neuroinflammation.

### Absence of ADAM8 reduces HMGB1-induced microglial neuroinflammation, proliferation, and migration.

**Fig. 3:**
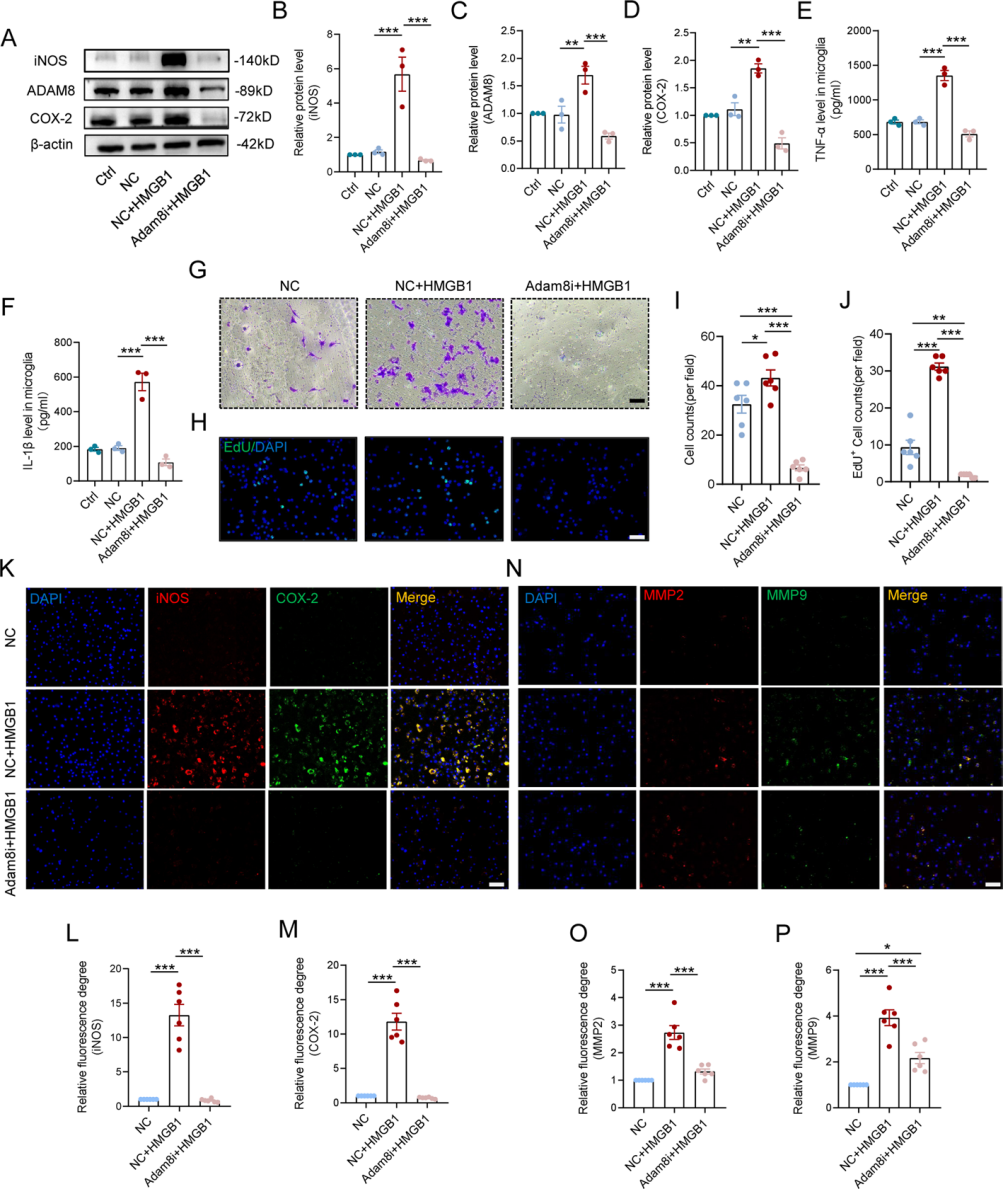
Knockdown ADAM8 reduce HMGB1-induced microglial neuroinflammation, proliferation, and migration. **A–D** Knockdown of ADAM8 in microglial cells suppresses the increase in iNOS (**B**) and COX-2 (**D**) induced by HMGB1 treatment. Quantitative analysis of ADAM8 (**C**) protein level in HMGB1 treatment and with or without ADAM8-knockdown microglia. **E**, **F** The levels of IL-1β (**E**) and TNF-α (**F**) in HMGB1 treatment and with or without ADAM8-knockdown microglia by ELISAs. **G** Knockdown ADAM8 reduce HMGB1-induced the migration of microglia by Transwell assay. n = 6. Scale bar = 100 μm. **H** Double immunofluorescence labeling of microglia for EdU(green)/ DAPI (blue). n = 6. Scale bar = 100 μm. **I** Quantitative analysis of **G**. **J** Quantitative analysis of **H**. **K, N** Triple immunofluorescence labeling of microglia for iNOS (red)/ COX-2 (green) / DAPI (blue) and MMP2 (green)/ MMP9 (red) / DAPI (blue). n = 6. Scale bar = 50 μm. **L–P** Quantitative analysis of iNOS, COX-2, MMP2 and MMP9 fluorescence level in with or without ADAM8-knockdown microglia. (*p < 0.05, **p < 0.01, and ***p < 0.001)

To further investigate the molecular function of ADAM8 in microglia-mediated neuroinflammation, we inhibited the expression of ADAM8 using shRNAs, which showed that inhibition of ADAM8 by the three shRNAs significantly inhibited the protein level of ADAM8 (Fig. S2A and S2B). Furthermore, HMGB1 treatment caused approximately a six-fold increase in the protein level of inducible nitric oxide synthase (iNOS) and a two-fold increase in cyclcoxygenase-2 (COX-2), which are two important inducer enzymes in the inflammatory response. However, we found that their expressions in ADAM8-knockdown microglia were inhibited by four to six times, and were even below normal levels (Fig. 3A, B, D), along with a significant decrease in the levels of ADAM8 protein (Fig. 3A, C). In addition, the degree of inflammation in microglia was reflected by the representative inflammatory cytokines interleukin (IL)-1β and tumor necrosis factor (TNF)-α; the results of ELISAs showed that the levels of IL-1β and TNF-α increased nearly three-fold in inflammatory microglia, but this increase was significantly reversed by ADAM8 knockdown (Fig. 3E, F). Furthermore, the levels of secretion of TNF-α decreased to below normal levels of the inflammatory microglia, which was inhibited by expression of ADAM8 (Fig. S2C). Because of the GO analysis data from RNA-seq, we also examined the levels of migration and proliferation when microglia were stimulated by HMGB1. The migration capacity of microglia during inflammation was measured using a Transwell assay, which showed that HMGB1 accelerated the migration of microglia, and this was almost completely inhibited during the absence of ADAM8 (Fig. 3G, I). Similarly, EdU positive microglia treated with HMGB1 were minimally affected when ADAM8 was inhibited (Fig. 3H, J). The level of inflammatory activation of microglia was also visualized by IF staining, showing that delivery of HMGB1 triggered excessive expressions of iNOS and COX-2 in microglia, which was restored to the level of the NC group (Fig. 3K–M). Moreover, we determined expressions of the inflammation-related matrix metalloproteinases (MMP) 2 and 9 produced by microglia. Both WB and IF staining showed a significant increase in the expressions of MMP 2 and 9 in HMGB1-treated microglia, while such increases were inhibited by the interference of ADAM8 expression (Fig. 3N–P, and S3A-S3C).

### The tail regions of ADAM8 and ERKs promote the phosphorylation and nuclear translocation of Fra-1

**Fig. 4:**
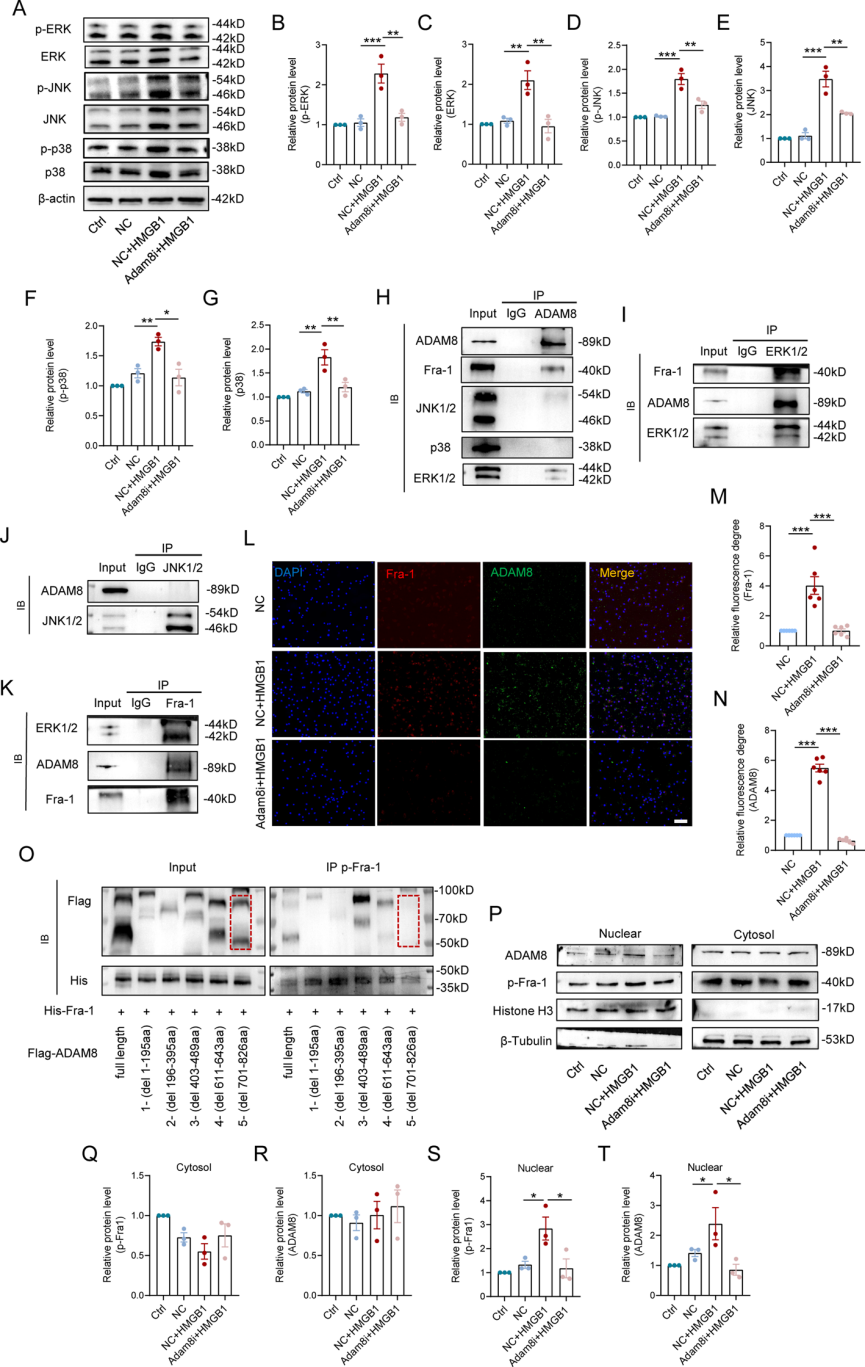
The tail regions of ADAM8 and ERKs facilitate the phosphorylation and nuclear translocation of Fra-1. **A **Western blotting performed for the protein level of ERK1/2, JNK1/2 and p38 in microglia treated with HMGB1 and subjected to ADAM8 knockdown or control conditions. β-acin was used as the control. **B–G** Quantitative analysis indicates an increase in the phosphorylation or total levels of ERK, JNK, and p38 proteins due to elevated HMGB1, which is negated upon ADAM8 knockdown. **H** CoIP of Fra-1, JNK1/2, p38 and ERK1/2 protein with ADAM8 in microglia. **I** CoIP of Fra-1 and ADAM8 protein with ERK1/2 in microglia. **J** CoIP of ADAM8 protein with JNK1/2 in microglia. **K** CoIP of ERK1/2 and ADAM8 protein with Fra-1 in microglia. **L** Triple immunofluorescence labeling of microglia for Fra-1 (red)/ADAM8 (green)/DAPI (blue). n = 6. Scale bar = 50 μm. **M, N** Quantitative analysis of Fra-1 and ADAM8 fluorescence level in with or without ADAM8-knockdown microglia. **O** CoIP of full-length and deletion (del) in five different regions of ADAM8 protein with p-Fra-1 in 293 T cells with Fra-1 overexpression. **P** Western blotting performed for the protein level of ADAM8 and Fra-1 in microglia treated with HMGB1 and subjected to ADAM8 knockdown or control conditions. Histone was used as the control in nuclear. β-tubulin was used as the control in cytosol. **Q–T** Quantitative analysis indicates an increase in the phosphorylation or total levels of Fra-1 and ADAM8 proteins in cytosol or nuclear due to elevated HMGB1, which is negated upon ADAM8 knockdown. SD is represented by the error bars. (*p < 0.05, **p < 0.01, and ***p < 0.001)

We next identified the underlying molecular mechanism of how ADAM8 positively regulated microglial inflammation. Previous evidence showed that ADAM8 was closely associated with MAPK signaling, resulting in regulation of migratory or proinflammatory phenotypes. Figure 4A shows the phosphorylation of the MAPKs signal proteins, including ERK1/2, JNK1/2 and p38, along with their phosphorylated forms using western blot assays. As expected, whether measuring the phosphorylated level or the total level, the levels of all three proteins increased in HMGB1-treated microglia; however, knockdown of ADAM8 suppressed the levels of these proteins to similar levels as the NC group (Fig. 4B–G). To ascertain whether ADAM8 regulated MAPK proteins by direct interaction, we performed IP assays. The results of the ADAM8 IP showed that ERK1/2 and Fra-1 proteins were co-immunoprecipitated with ADAM8, but not with JNK1/2 and p38 (Fig. 4H). The results of reverse IP showed that ERK1/2 bound to ADAM8, as well as Fra-1, but JNK1/2 was incapable of binding to ADAM8 (Fig. 4I, J). We also found that Fra-1 bound to ADAM8 and ERK1/2 (Fig. 4K), suggesting that ADAM8 regulated ERK1/2 and Fra-1 by a direct interaction. We also showed co-localization between ADAM8 and Fra-1 in HMGB1-sitimulated microglia, and knockdown of ADAM8 reduced co-localization and inhibited nuclear localization of Fra-1 (Fig. 4L–N). Western blots showed that the total and phosphorylated levels of Fra-1 were significantly reduced during knockdown of ADAM8 in microglia (Fig. S4A–C). We further transfected with amino acids (aa) sequence deletion (del) in five different regions of ADAM8 (Flag labeled), according to the uniport database (https://www.uniprot.org), into 293 T cells with Fra-1 overexpression (His labelled) to ascertain which domain of ADAM8 bound to Fra-1. The results of the IP assay showed that full-length, del of 1–195 aa, del of 196–395 aa, del of 403–489 aa, and del of 611–643 aa were linked to phosphorylated Fra-1, but only del of 701–826 aa lost the ability to bind phosphorylated Fra-1 (Fig. 4O), suggesting that ADAM8 bound to Fra-1 by its 701–826 aa to promote Fra-1 phosphorylation. After distinguishing between cytosolic protein and nucleoprotein, we also found that ADAM8 entered into the nucleus and increased the expression of nuclear p-Fra-1, which was inhibited when the microglia were ADAM8i (Fig. 4P–T). Taken together, the results further indicated that ADAM8 regulated the activation and nuclear translocation of Fra-1 by direct binding.

### Fra-1 facilitates microglial activities by up-regulating the transcription of Map3k4 during neuroinflammation

**Fig. 5:**
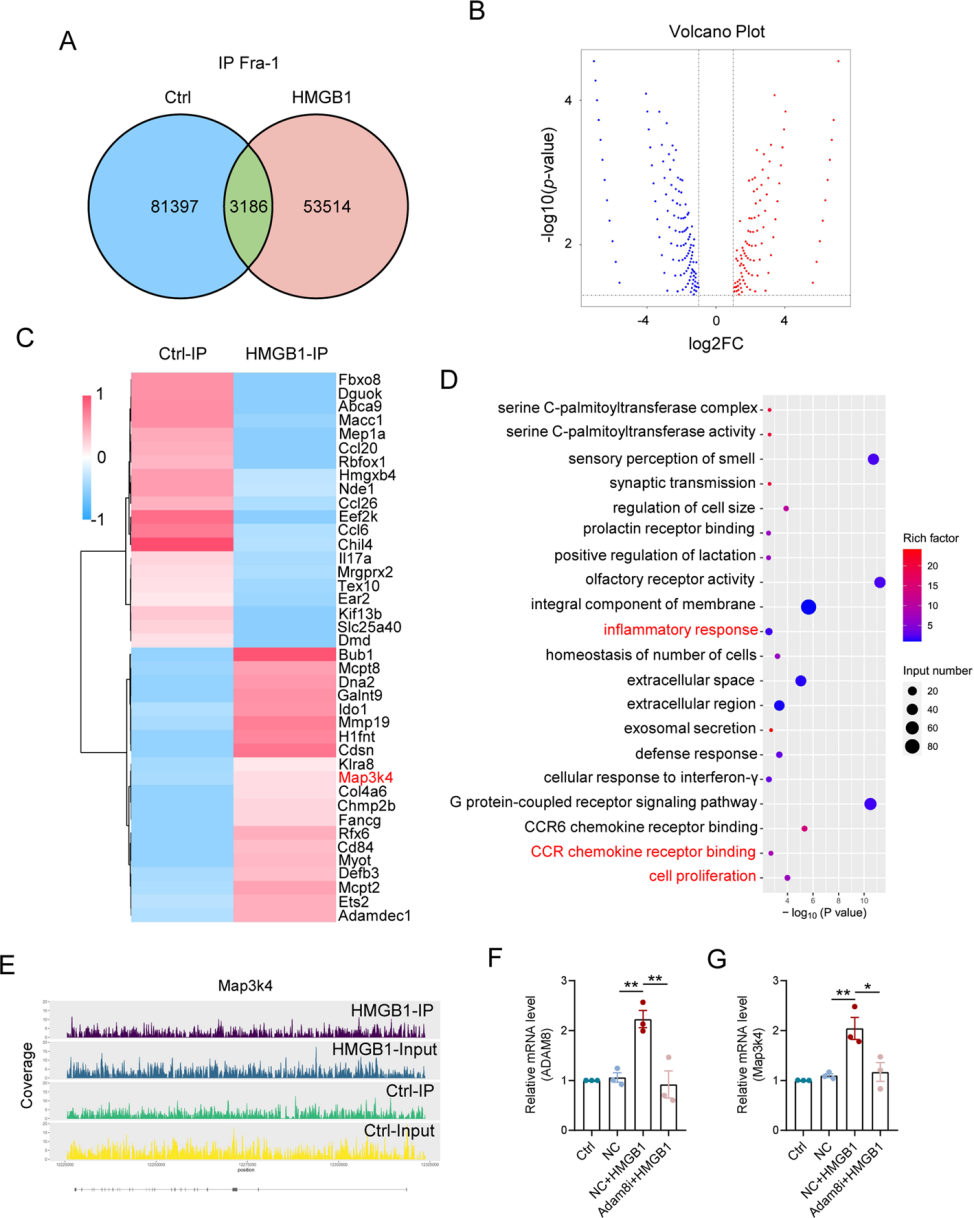
Fra -1 boosts microglial activities in neuroinflammation through the upregulation of Map3k4 transcription. **A** Identification of downstream target genes of Fra-1 in both the Ctrl and HMGB1 groups via ChIP-Seq. **B** Volcano plots depicting genes significantly upregulated and downregulated upon HMGB1 treatment compared to the Ctrl group. **C** A heat map displaying 20 upregulated and 20 downregulated genes associated with inflammation, migration, and proliferation. **D** GO analysis revealed differentially expressed peaks between the HMGB1 and Ctrl groups. **E** Signal intensities of sequencing across DNA segments of the Map3k4 gene following microglial HMGB1 stimulation. **F, G** Quantitative analysis of the mRNA levels of ADAM8 (**F**) and Map3k4 (**G**) in HMGB1 treatment and with or without ADAM8-knockdown microglia (*p < 0.05, **p < 0.01, and ***p < 0.001)

To better explain how ADAM8 regulated the activation of JNK1/2 and p38 signals, we speculated that Fra-1, as a member of the Fos transcription factor family, regulated some key gene expressions in HMGB1-treated microglia. We used ChIP-Seq to screen the downstream target genes of Fra-1. There were 3,186 genes regulated by Fra-1, which were differentially expressed both in the Ctrl and HMGB1 groups (Fig. 5A). Volcano plots showed that HMGB1 treatment significantly up-regulated 179 genes and down-regulated 196 genes, when compared with the Ctrl group (Fig. 5B). We screened 20 up-regulated genes and 20 down-regulated genes related to inflammation, migration, and proliferation, and displayed them in a heat map, where Map3k4 was a positive up-stream regulator for the activation of MAPK pathways (Fig. 5C). Compared with the Ctrl group, the GO analysis showed that peaks differentially expressed in the HMGB1 group were closely involved in inflammatory response, CCR chemokine receptor binding, and cell proliferation (Fig. 5D), suggesting that Fra-1 functioned as a crucial transcription factor in microglia during HMGB1-induced inflammation. As shown in Fig. 5E, Fra-1 regulated the intensities of sequencing signals on each DNA segment of the *Map3k4* gene after HMGB1 stimulation in microglia. We then further verified the mRNA level of Map3k4 in HMGB1-stimulated microglia after ADAM8i. As expected, our results showed that the expression of Map3k4 was significantly reduced when ADAM8 was successfully inhibited in inflammatory microglia (Fig. 5F, G), suggesting that ADAM8 promoted the activation of JNK1/2 and p38 via the Fra-1/Map3k4 axis.

### Pharmacological inhibition of ADAM8 alleviates neuroinflammation by reducing Fra-1 expression in SCI mice

**Fig. 6:**
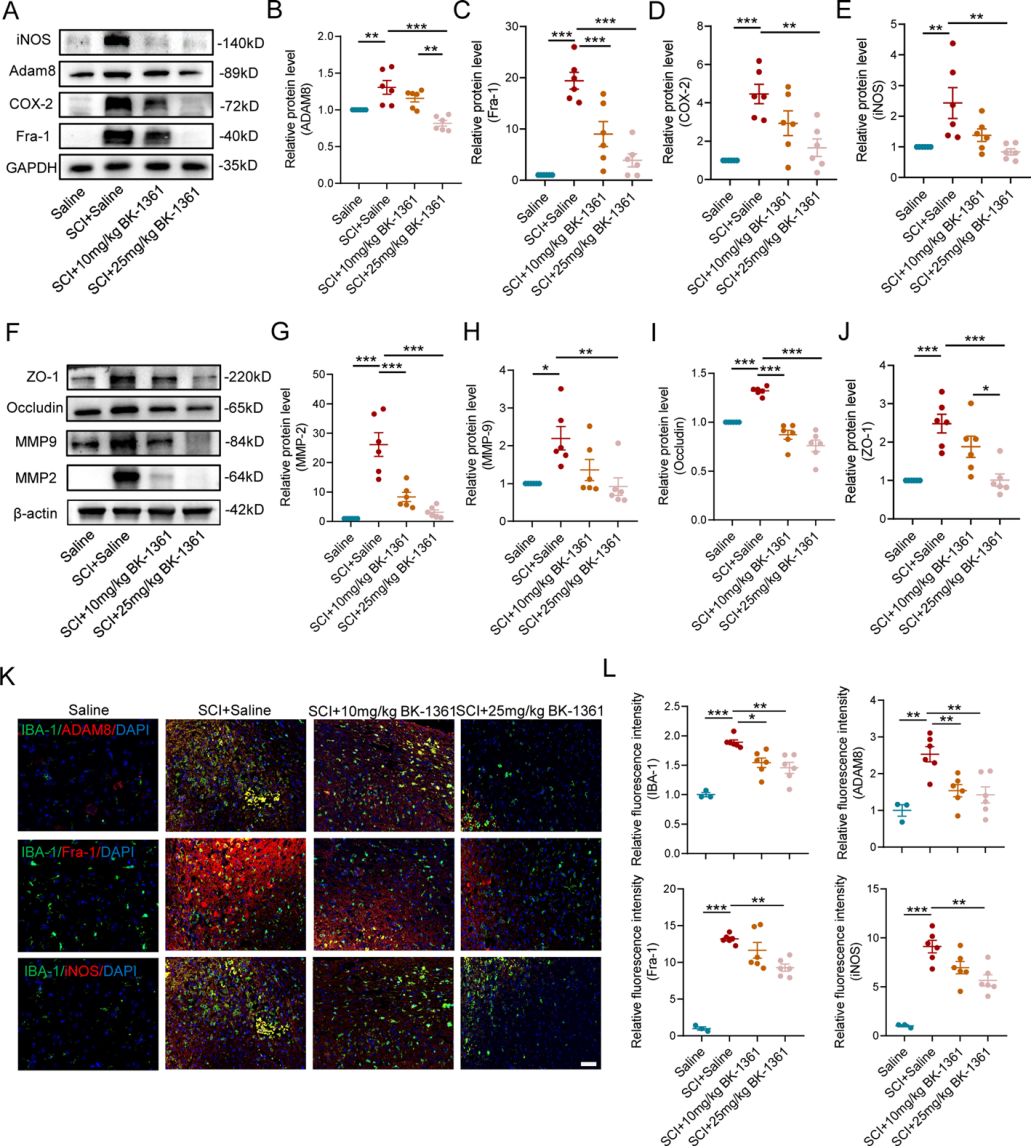
Alleviation of neuroinflammation through BK-1361 inhibition of ADAM8 involves the downregulation of Fra-1 expression in SCI mice. **A** Western blotting performed for the protein level of iNOS, ADAM8, COX-2, and Fra-1 in mice treated with low and high doses of BK-1361 or control conditions. GAPDH was used as the control. **B–E** Quantitative analysis of the protein level of ADAM8 (**B**), Fra-1 (**C**), COX-2 (**D**) and iNOS (**E**) in mice treated with low and high doses of BK-1361 or control conditions. **F** Western blotting performed for the protein level of ZO-1, occludin, MMP2 and MMP9 in mice treated with low and high doses of BK-1361 or control conditions. β-actin was used as the control.** G–I** Quantitative analysis of the protein level of MMP2 (**G**), ZO-1 (**H**), occludin (**I**) and MMP9 (**J**) in mice treated with low and high doses of BK-1361 or control conditions. **K** Triple immunofluorescence labeling of in SCI mice treated with low and high doses of BK-1361 or control conditions for IBA-1 (green)/ADAM8 (red)/DAPI (blue), IBA-1 (green)/Fra-1 (red)/ DAPI (blue) or IBA-1 (green)/iNOS (red)/DAPI (blue). n = 6. Scale bar = 50 μm.** L** Quantitative analysis of IBA-1, Fra-1 ADAM8 and iNOS fluorescence level in SCI mice treated with low and high doses of BK-1361 or control conditions (*p < 0.05, **p < 0.01, and ***p < 0.001)

We next used BK-1361, a specific inhibitor of ADAM8, to determine whether inhibition of ADAM8 alleviated neuroinflammation after SCI. Before the SCI mice were treated with BK-1361, we injected 10 mg/kg and 25 mg/kg BK-1361 into healthy mice for 7 days. HE staining showed that the mouse spinal cord, brain, heart, liver, spleen, lung, and kidney did not show obvious damage (Fig. S5), indicating that administration of low and high doses of BK-1361 in mice was safe. At dpi 3, we found that the level of ADAM8 significantly decreased after BK-1361 administration in SCI mice, especially at a dose of 25 mg/kg (Fig. 6A, B). In addition, the expression of Fra-1 was reduced along with decreased expression of ADAM8 in BK-1361-treated mice (Fig. 6A, C). For inflammatory mediators, we determined the expressions of iNOS and COX-2, showing that their levels were inhibited after either 10 mg/kg or 25 mg/kg BK-1361 treatment (Fig. 6A, D, E). Considering the important effects of MMPs on secondary SCI and neuroinflammation, the levels of MMP2 and MMP9 were measured, which showed that both were significantly decreased at a dose of 25 mg/kg BK-1361 treatment in SCI mice (Fig. 6F–H). Unexpectedly, the western blot results also showed a decrease in tight junction proteins, including zonula occludin 1 (ZO-1), in both the 10 and 25 mg/kg BK-1361-treated mice (Fig. 6F. I, J). We also found a decrease in the number of microglia surrounding the injured foci, along with reduced expressions of ADAM8 and Fra-1 in microglia following concentration-dependent BK-1361 treatments in the SCI mice (Fig. 6K, L). Overall, the results showed that the expression of iNOS in microglia significantly decreased after treatment with 25 mg/kg BK-1361 in SCI (Fig. 6K, L).

### Administration of ADAM8 inhibitor decreases glial scarring and axon demyelination after SCI

**Fig. 7:**
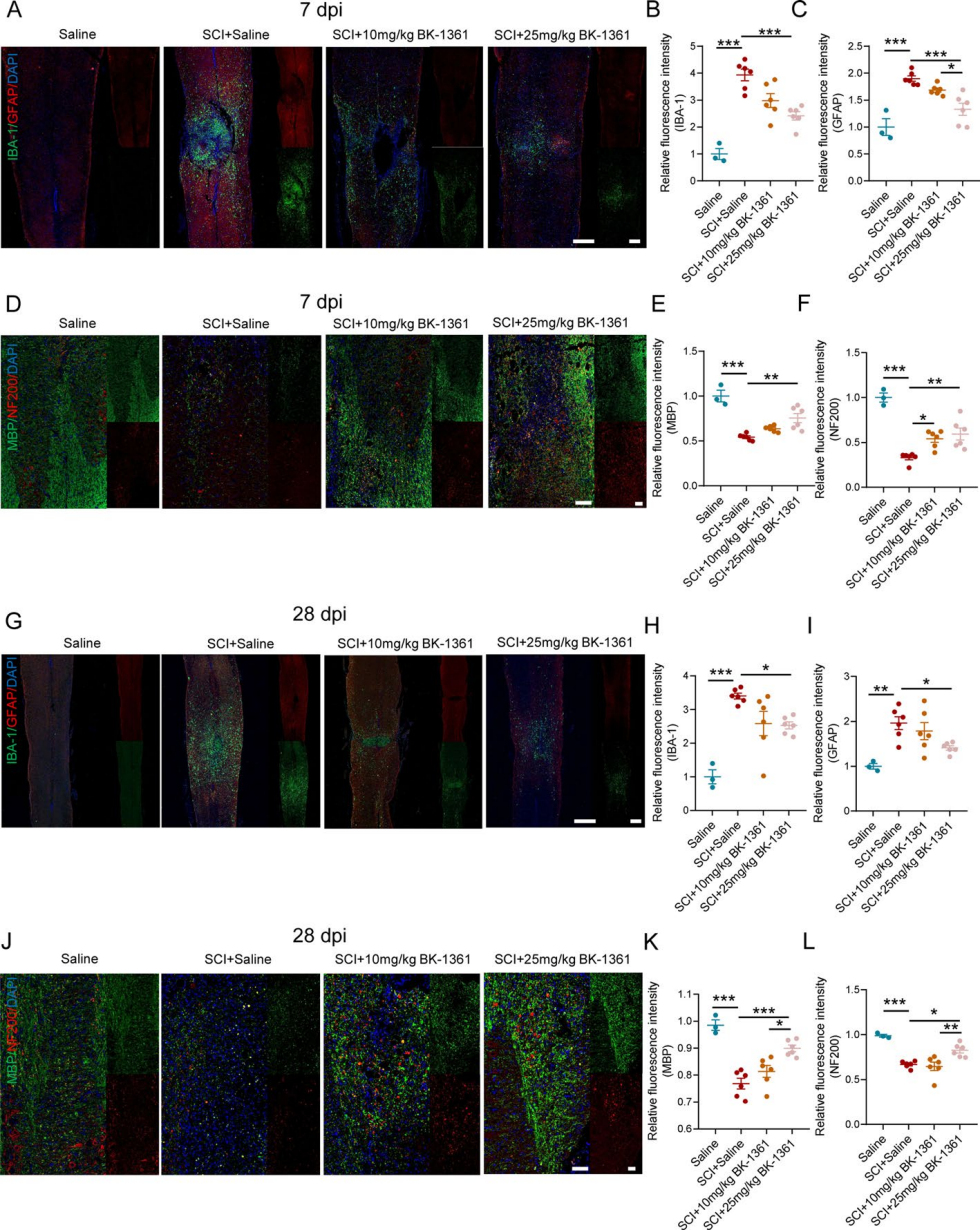
Treatment with ADAM8 inhibitor reduces glial scarring and axon demyelination post SCI. **A** Immunofluorescent triple labeling of IBA-1 (green)/GFAP (red)/DAPI (blue) in SCI mice after 3 days of treatment with low and high doses of BK or under control conditions. n = 6. Scale bar = 500 μm. **B**, **C** Quantitative analysis of IBA-1 and GFAP fluorescence level in SCI mice after 3 days of treatment with low and high doses of BK or under control conditions. **D** Immunofluorescent triple labeling of MBP (green) / NF200 (red)/ DAPI (blue) in SCI mice after 3 days of treatment with low and high doses of BK or under control conditions. n = 6. Scale bar = 100 μm. **E**, **F** Quantitative analysis of MBP and NF200 fluorescence level in SCI mice after 3 days of treatment with low and high doses of BK or under control conditions. **G** Immunofluorescent triple labeling of IBA-1 (green)/GFAP (red)/DAPI (blue) in SCI mice after 28 days of treatment with low and high doses of BK or under control conditions. n = 6. Scale bar = 500 μm. **H**, **I** Quantitative analysis of IBA-1 and GFAP fluorescence level in SCI mice after 28 days of treatment with low and high doses of BK or under control conditions. **J** Immunofluorescent triple labeling of MBP (green) / NF200 (red)/ DAPI (blue) in SCI mice after 28 days of treatment with low and high doses of BK or under control conditions. n = 6. Scale bar = 100 μm. **K**, **L** Quantitative analysis of MBP and NF200 fluorescence level in SCI mice after 28 days of treatment with low and high doses of BK or under control conditions (*p < 0.05, **p < 0.01, and ***p < 0.001)

We next determined whether BK-1361 treatment resulted in improvement in glial scarring and axon demyelination when it effectively inhibited neuroinflammation. At 7 dpi, we found that dominating IBA-1 positive microglia-macrophages accumulated in the injured foci, along with surrounding subordinate astrocytes, while treatment with BK-1361, especially at 25 mg/kg, decreased the number of microglia-macrophages and astrocytes (Fig. 7A–C). In addition, the distributions of the myelin sheath and axons were visualized by IF staining, which showed that SCI caused a loss of myelin structure, axon connection, and myelin-coating axons, but treatment with BK-1361 increased the area of myelin, the number of axons, and myelin-coating axons (Fig. 7D–F). At 28 dpi, the glial scar was comprised of large amounts of astrocytes and small amounts of microglia-macrophages, aggregated around the injury epicenter, but there were less astrocytes and microglia-macrophages surrounding the injury foci in the BK-1361-treated SCI mice than those in the saline-treated SCI mice (Fig. 7G–I). We also found large-scale disruption of myelin structures and of neurons and axons after SCI (Fig. 7J–L). Furthermore, treatment with 25 mg/kg BK-1361 increased the area of myelination and also promoted the amount of axon regeneration, although it did not show an effect at a dose of 10 mg/kg (Fig. 7J–L). Together, these results suggested that treatment with BK-1361 at 25 mg/kg may be an effective dose for improvement of secondary post-SCI neuropathies.

### Inhibition of ADAM8 attenuates disruption of neurohistology and dysfunction of hindlimb locomotor functions after SCI

**Fig. 8:**
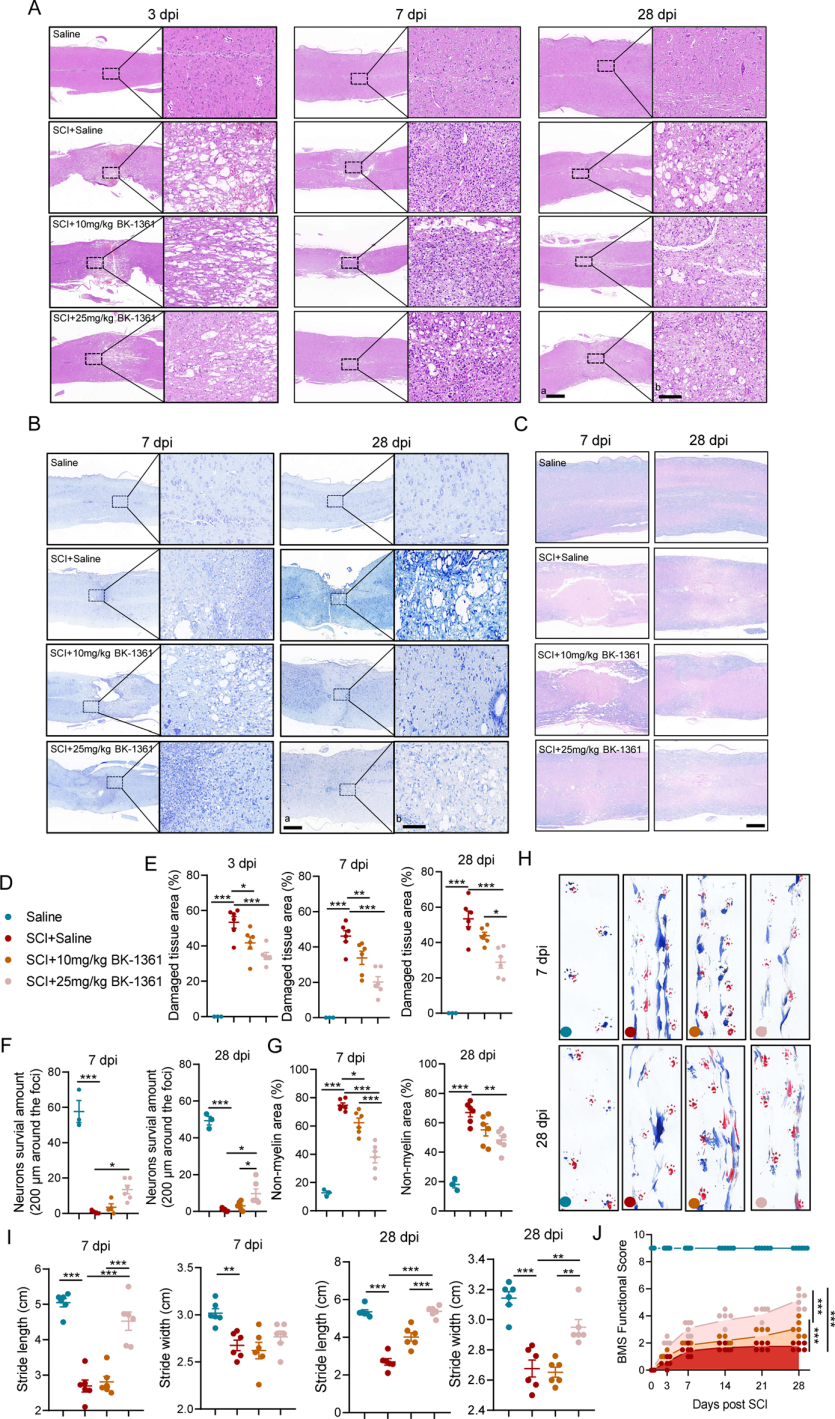
Administration of ADAM8 inhibitor mitigates disruption in neurohistology and improves hindlimb locomotor function following SCI.** A** Representative images for HE staining obtained from longitudinal sections centered around the injured core at 3, 7 and 28 dpi in SCI and low and high doses of BK-1361 treatment SCI mice; Scale bar: a = 500 μm, b = 20 μm. **B** Representative images for Nissl staining obtained from longitudinal sections centered around the injured core at 7 and 28 dpi in SCI and low and high doses of BK-1361 treatment SCI mice; Scale bar: a = 500 μm, b = 20 μm. **C** Representative images for LFB staining obtained from longitudinal sections centered around the injured core at 7 and 28 dpi in SCI and low and high doses of BK-1361 treatment SCI mice; Scale bar = 500 μm. **D** This part of the experiment is divided into 4 groups, saline (blue), SCI + saline (red), SCI + 10 mg/kg BK-1361 (dark blue or 2) and SCI + 25 mg/kg BK-1361 (pink). **E** Quantitative analysis of damaged tissue area in SCI mice after 3, 7 and 28 days of treatment with low and high doses of BK-1361 or under control conditions. n = 6. **F** Quantitative analysis of the neuronal survival amount (200 μm around the foci) in SCI mice after 7 and 28 days of treatment with low and high doses of BK-1361 or under control conditions. n = 6. **G** Quantitative analysis of the non- myelin area in SCI mice after 7 and 28 days of treatment with low and high doses of BK-1361 or under control conditions. **H** Representative images for walking track assay at 7 and 28 dpi in SCI and low and high doses of BK-1361 treatment SCI mice. **I** Quantitative analysis of the walking track assay in SCI mice after 7 and 28 days of treatment with low and high doses of BK-1361 or under control conditions. n = 6. **J** Assessment of hind limb locomotor recovery was conducted via the BMS open-field test. n = 6. SD is represented by the error bars (*p < 0.05, **p < 0.01, and ***p < 0.001)

To better validate the histological and locomotor functional improvements of pharmacological inhibition of ADAM8 after SCI, we further performed histological staining, including HE, Nissl and LFB staining, as well as locomotor evaluation, such as footprint assays using the BMS. There were decreases in hemorrhages and infiltration of peripheral cells at 3 and 7 dpi (Fig. 8A, S6A, and S6B), and tissue defects at 3, 7, and 28 dpi with an increase of BK-1361 concentrations (Fig. 8A, E). Nissl staining showed that treatment with BK-1361 significantly increased the amount of neuronal survival, at 7 dpi or 28 dpi (Fig. 8B, F). We also reconfirmed that BK-1361 protected myelin structures from neuroinflammation-mediated collapse, owing to the preservation of LFB positive areas, both at 7 and 28 dpi (Fig. 8C, G). For behavioral assessments, we found an increase in stride length of hind limbs in the 25 mg/kg BK-1361-treated SCI mice at 7 and 28 dpi, but only an improvement in stride width of hind limbs in the SCI mice with 25 mg/kg at 28 dpi (Fig. 8H, I). Furthermore, BMS showed that the scores involving treatment with 25 mg/kg BK-1361 were significantly higher than those obtained from other SCI mice, which began at 3 dpi and 21 dpi, when scores from mice treated with 10 mg/kg BK-1361 administration were significantly higher than those from the saline-treated SCI mice (Fig. 8J). Overall, treatment with either dose of BK-1361 significantly improved BMS in mice after SCI.

## Discussion

Improving neuropathology during SCI by regulating critical molecules in microglia-mediated neuroinflammation is a viable approach, but currently there is no effective method for anti-inflammatory therapy, except steroid pulse therapy, with minimal efficacy [37, 38]. In the present study, we showed that ADAM8, as a critical molecule positively regulating neuroinflammatory development and secondary pathogenesis, promoted microglial activation, migration, and proliferation during neuroinflammation by forming a complex with ERK and Fra-1, to further activate the Map3k4/JNK/p38 axis. Furthermore, inhibition of ADAM8 by administration of BK-1361 attenuated the level of neuroinflammation, glial formation, and neurohistological loss, leading to favorable improvement in locomotor functional recovery in SCI mice.

Genetic up-regulation of ADAM8 has been previously reported in the injured sciatic nerve of mice, resulting in regulation of the cell adhesion and extracellular matrix (ECM) degradation [39]. A recent study [40] reported that ADAM8 was linked to neuroinflammation-induced autism spectrum disorder in children. Our results also showed that ADAM8 was found in microglia and neurons, and mRNA-Seq data showed that its expression increased in microglia after SCI, suggesting that increased ADAM8 expression was closely related to an inflammatory response mediated by microglia. In neurodegeneration of brain and spinal cord, an increase in ADAM8 expression was found in response to TNF-α; however, our findings showed that ADAM8 was released by injured neurons, which shed TNFR1 to block TNFα-stimulated death signaling [41], indicating that ADAM8 played a neuroprotective role in injured neurons. To avoid decreasing ADAM8’s protective effect on neurons, we first injected BK-1361 into mice at 6 h after SCI.

ADAM8 is a proteolytic enzyme involved in cleavage of various precursor proteins, signal receptors, and ECMs into their soluble forms, but its role in intracellular signal transduction has not been reported in central nervous system diseases and neurocytes during neuroinflammation. Notably, the role of ADAM8 in inflammation has been investigated in multiple diseases of the respiratory system, such as ARDS [31], allergic asthma [33], and acute pneumonia [42], which all showed that ADAM8 promoted leukocyte recruitment, to exacerbate airway inflammation. However, few studies have reported the specific molecular mechanism of ADAM8 action. After we ascertained that increased ADAM8 was specifically located in microglia after SCI, we further found that inhibition of ADAM8 reduced inflammatory levels, as well as microglia recruitment and proliferation. Considering that ADAM8 critically promoted proliferation and invasion of cancer cells related to MAPKs signaling [43, 44], we determined whether ADAM8 promoted neuroinflammatory activities in microglia by directly acting on ERK1/2, JNK1/2 or p38. Although we found remarkable decreases in the expressions and phosphorylations of ERK1/2, JNK1/2, and p38 after inhibition of ADAM8, only ERK1/2 was capable of interacting directly with ADAM8. This is consistent with a previous study, which reported increased expression of the MAPK signaling pathway with differentially expressed genes of IL-1β and ADAM8 in particulate matter-induced inflammatory lung injury [45]. Therefore, we also carefully examined how ADAM8 affected JNK1/2 and p38.

Fosl-1, encoding Fra-1, is a crucial member of the AP-1 transcription factor family involved in inflammatory development [46], where its expression has been associated with secondary SCI progression [47, 48]. Importantly, Fosl-1 induced apoptosis and neuroinflammation, especially in neurons and microglia, have been shown during SCI [48]. We also provided evidence that Fosl-1 and Fra-1 were highly expressed after SCI and during resulting neuroinflammation. Activated phospho-ERK1/2 is capable of stabilizing Fra-1 phosphorylation and promoting nuclear translocation, to bind with gene promoters [49–51]; however, once Fra-1 is dephosphorylated, it is rapidly degraded after ubiquitination or sumoylation [52–54]. Our results showed decreased expressions of Fra-1 and ERK1/2 proteins, along with their phosphorylated forms, when ADAM8 was inhibited in microglia, where the C-terminal tail of ADAM8 bound with Fra-1. This suggested that this region of ADAM8 may interact with ERK1/2 and Fra-1 to form a tripolymer, preventing Fra-1 from inactivation. The observation that ADAM8 was found in the nucleus further supported this possibility. The activation of Fra-1 enhanced the inflammatory response by reducing a M2-like type of macrophage polarization [55], which increased MMP-9 secretion [56], and aggravated cytokine production [57]. Using a ChIP-seq screening assay, we found that Fra-1 reactivated Map3k4. Map3k4 is an activated MAPK kinase kinase, which activates JNK and p38 using a specific MAPK kinase [58, 59]. As expected, expression of Map3k4 occurred with inhibition of ADAM8 in microglia during inflammation, suggesting that ADAM8 activated JNK1/2 and p38 signaling using the Fra-1/Map3k4 axis.

Our previous studies and the results of studies of others have demonstrated that effectively inhibiting the MAPK signaling pathway during the early phase of secondary SCI mitigates neuroinflammation-mediated neuropathies and improves locomotor functional recovery [60–64]. However, current specific inhibitors targeting the MAPK signaling pathway cannot be translated into clinical therapy due to obvious cytotoxic reactions, so it is a feasible strategy to identify other novel specific inhibitors with low toxicity and strong inhibition to replace the original MAPK inhibitors for clinical treatments. BK-1361 is a peptidomimetic ADAM8 inhibitor, affecting ADAM8 function, and leading to less ERK1/2 and MMP activation, with an anti-invasion effect in pancreatic cancer [65]. In addition, BK-1361 also can alleviate bronchial hyperresponsiveness in asthma [66], but its potential in the treatment of SCI remains unclear. After confirming that the dose of BK-1361 did not cause vital organ damage, we tested its effect in the treatment of SCI mice. Our results showed a decrease in neurohistological disruption, glial scarring, and locomotor dysfunction in the SCI mice treated with BK-1361. This effect was the result of the potent anti-neuroinflammation effect of ADAM8 inhibition by suppressing Fra-1 activation. However, we found a decrease in MMP2/9 associated with a reduction of ZO-1 and occludin. Theoretically, decreased MMP2/9 expression may cause less destruction of tight junctions, with increased expressions of ZO-1 and occludin. This result may explain the inhibitory effect of ADAM8 inhibition on angiogenesis [67] or glial accumulation.

Although the current study did not identify whether ADAM8 promoted Fra-1 activation, which depended on ERK1/2 expression, we further verified the important pathogenic role of ADAM8 after SCI by using a conditional ADAM8 knockout mouse, targeting microglia, and demonstrated a previously unreported intranuclear, rather than extracellular, function of ADAM8 in microglia-mediated neuroinflammation. By directly regulating the Fra-1/Map3k4/MAPKs axis, the potent pharmacological action of the ADAM8 inhibitor, BK-1361 improved neuroinflammatory suppression and neurofunction. In the future, we will continue to characterize other potential mechanisms of ADAM8 in the regulation of neuroinflammation, and we plan to comprehensively evaluate the pharmacological effects and unknown side effects of BK-1361 in the anti-inflammatory treatment of SCI.

## Conclusions

Our study suggested that ADAM8 was a critical molecule, which positively regulated neuroinflammatory development and secondary pathogenesis by promoting microglial activation and migration. Mechanically, ADAM8 formed a complex with ERK and Fra-1 to further activate the Map3k4/JNK/MAPK axis in microglia. Inhibition of ADAM8 by treatment with BK-1361 decreased the levels of neuroinflammation, glial formation, and neurohistological loss, leading to favorable improvement in locomotor functional recovery in SCI mice.

### Supplementary Information

Supplementary Material 1: Fig. S1. The protein level of ADAM8 in microglia treated with HMGB1 was peaked at 9 h. A Western blotting performed for the protein level of ADAM8 in microglia treated with HMGB1 from 0 to 24 h; n = 3. GAPDH was used as the control. B Quantitative analysis of ADAM8 expression. *p < 0.05 vs. 0 h (B) by one-way ANOVA followed by Tukey's post hoc analysis (*p < 0.05,**p < 0.01, and ***p < 0.001).

Supplementary Material 2: Fig. S2. Knockdown ADAM8 reduce HMGB1-induced TNF-α in microglial. A-B The three shRNAs significantly inhibited the protein level of ADAM8. β-acin was used as the control. C The levels of TNF-α in HMGB1 treatment and with or without ADAM8-knockdown microglia by ELISAs. *p < 0.05 vs. ctrl group (B and C) by one-way ANOVA followed by Tukey's post hoc analysis (*p < 0.05,**p < 0.01, and ***p < 0.001).

Supplementary Material 3: Fig. S3. Knockdown ADAM8 reduce HMGB1-induced MMP 2 and 9 protein levels in microglial. A Western blot analysis was performed to evaluate MMP 2 and 9 protein levels in microglia treated with HMGB1 and subjected to ADAM8 knockdown or control conditions. β-acin was used as the control. B-C Quantitative analysis of MMP 2 and 9 protein levels. (*p < 0.05, **p < 0.01, and ***p < 0.001).

Supplementary Material 4: Fig. S4. Knockdown ADAM8 reduce HMGB1-induced the total and phosphorylated levels of Fra-1 protein in microglial. A Western blot analysis was performed to evaluate the total and phosphorylated levels of Fra-1 protein in microglia treated with HMGB1 and subjected to ADAM8 knockdown or control conditions. β-acin was used as the control. B-C Quantitative analysis of the total and phosphorylated levels of Fra-1 protein. (*p < 0.05, **p < 0.01, and ***p < 0.001).

Supplementary Material 5: Fig. S5. HE staining revealed no apparent damage in the mouse spinal cord, brain, heart, liver, spleen, lung, or kidney following treatment with low and high doses of BK-1361. Scale bar: a = 200 μm, b = 50 μm, c = 1 mm, d = 100 μm Fig. S6 Quantitative analysis of the infiltrating cell counting at 3(A) and 7(B) dpi of Fig. 8A.

Supplementary Material 6: Fig. S6. Quantitative analysis of the infiltrating cell counting at 3(A) and 7(B) dpi of Fig. 8A.

Supplementary Material 7: Table.S1. Baseline Characteristics of the Patients.

## Data Availability

The data that support the findings of this study are available from the corresponding author upon reasonable request.

## Abbreviations

SCI: Spinal cord injury
DAMPs: Damage-associated molecular patterns
HMGB1: High mobility group box-1 protein
ADAM8: A disintegrin and a metalloproteinase domain 8
ARDS: Acute respiratory distress syndrome
COPD: Chronic obstructive pulmonary disease
PBS: Phosphate-buffered saline
FBS: Fetal bovine serum
DMEM: Dulbecco’s Modified Eagle Medium
GO: Gene Ontology
KEGG: Kyoto Encyclopedia of Genes and Genomes
ADAM8i: ADAM8 mRNA interference
WB: Western blotting
qRT-PCR: Real-time quantitative reverse transcription PCR
PVDF: Polyvinylidene fluoride
ChIP-Seq: Chromatin immunoprecipitation sequencing
PFA: Paraformaldehyde
IF: Immunofluorescence
HE: Hematoxylin–eosin
LFB: Luxol Fast Blue
BMS: Basso Mouse Scale
UMAP: Uniform manifold approximation and projection
CRP: C-reaction protein
COX-2: Cyclcoxygenase-2
MMP: Matrix metalloproteinases
ZO-1: Zonula occludin 1
ECM: Extracellular matrix
