# Supplementary figures and images for: Blockade of the ADAM8-Fra-1 complex attenuates neuroinflammation by suppressing the Map3k4/MAPKs axis after spinal cord injury
Source: Cell Mol Biol Lett. 2024 May 16;29:75. doi: 10.1186/s11658-024-00589-3 (PMC11100242; doi:10.1186/s11658-024-00589-3)

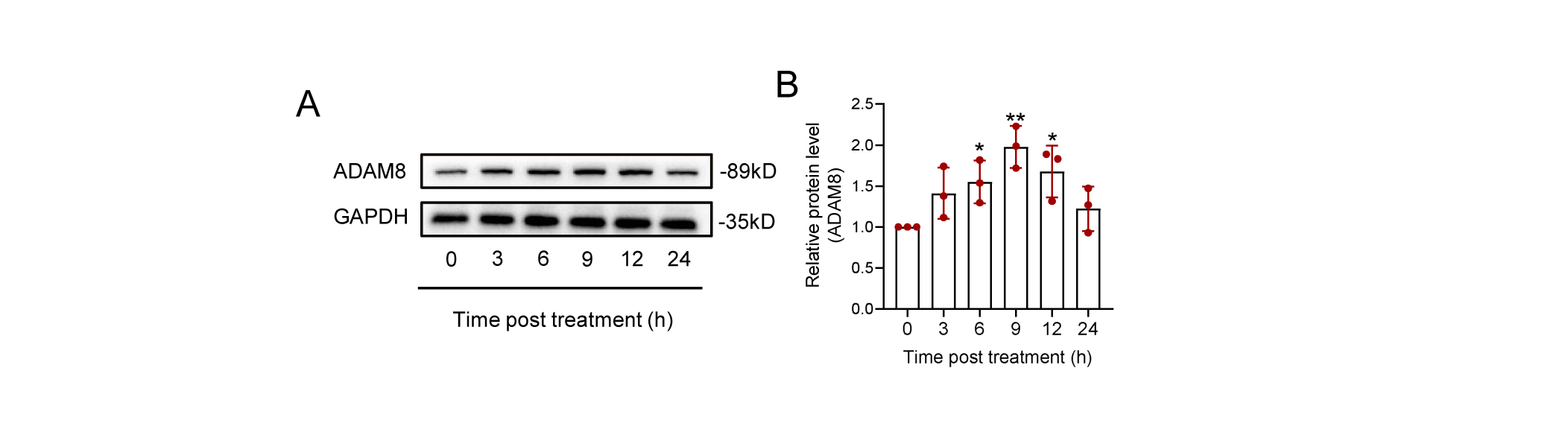

Supplement: Supplementary file 1 — Supplementary Material 1: Fig. S1. The protein level of ADAM8 in microglia treated with HMGB1 was peaked at 9 h. A Western blotting performed for the protein level of ADAM8 in microglia treated with HMGB1 from 0 to 24 h; n = 3. GAPDH was used as the control. B Quantitative analysis of ADAM8 expression. *p < 0.05 vs. 0 h (B) by one-way ANOVA followed by Tukey's post hoc analysis (*p < 0.05,**p < 0.01, and ***p < 0.001). [file 11658_2024_589_MOESM1_ESM.tif]

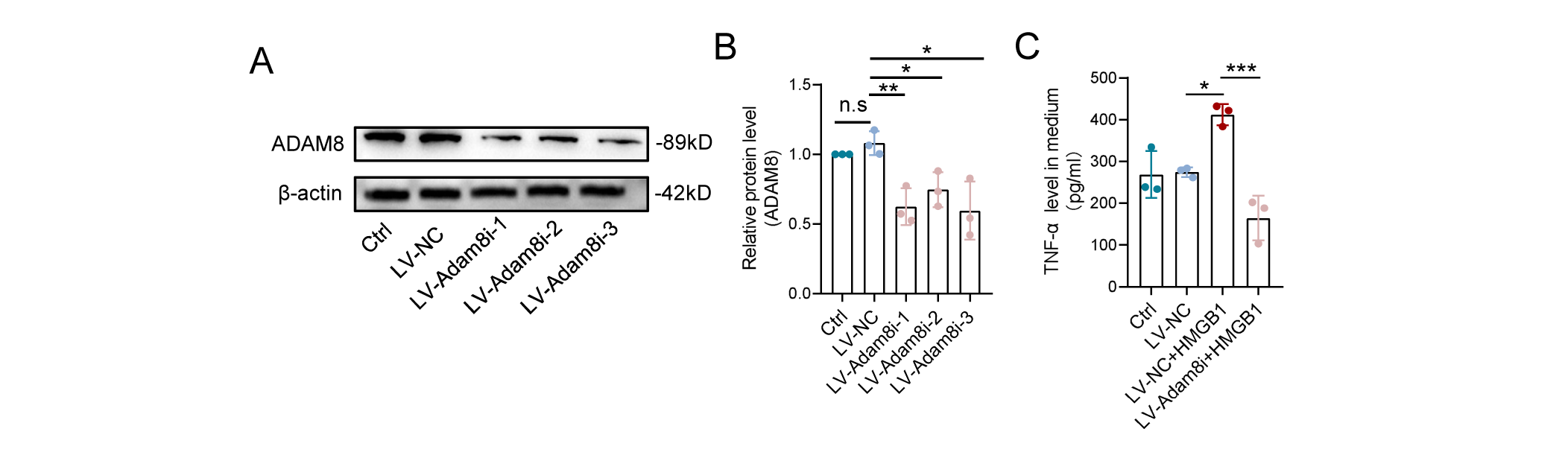

Supplement: Supplementary file 2 — Supplementary Material 2: Fig. S2. Knockdown ADAM8 reduce HMGB1-induced TNF-α in microglial. A-B The three shRNAs significantly inhibited the protein level of ADAM8. β-acin was used as the control. C The levels of TNF-α in HMGB1 treatment and with or without ADAM8-knockdown microglia by ELISAs. *p < 0.05 vs. ctrl group (B and C) by one-way ANOVA followed by Tukey's post hoc analysis (*p < 0.05,**p < 0.01, and ***p < 0.001). [file 11658_2024_589_MOESM2_ESM.tif]

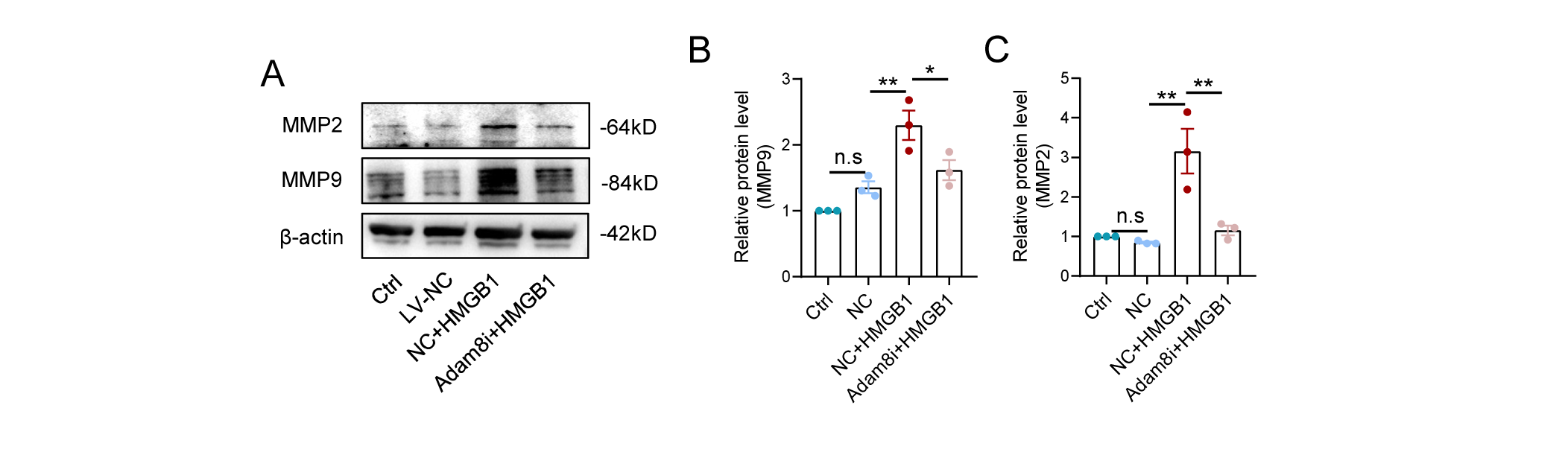

Supplement: Supplementary file 3 — Supplementary Material 3: Fig. S3. Knockdown ADAM8 reduce HMGB1-induced MMP 2 and 9 protein levels in microglial. A Western blot analysis was performed to evaluate MMP 2 and 9 protein levels in microglia treated with HMGB1 and subjected to ADAM8 knockdown or control conditions. β-acin was used as the control. B-C Quantitative analysis of MMP 2 and 9 protein levels. (*p < 0.05, **p < 0.01, and ***p < 0.001). [file 11658_2024_589_MOESM3_ESM.tif]

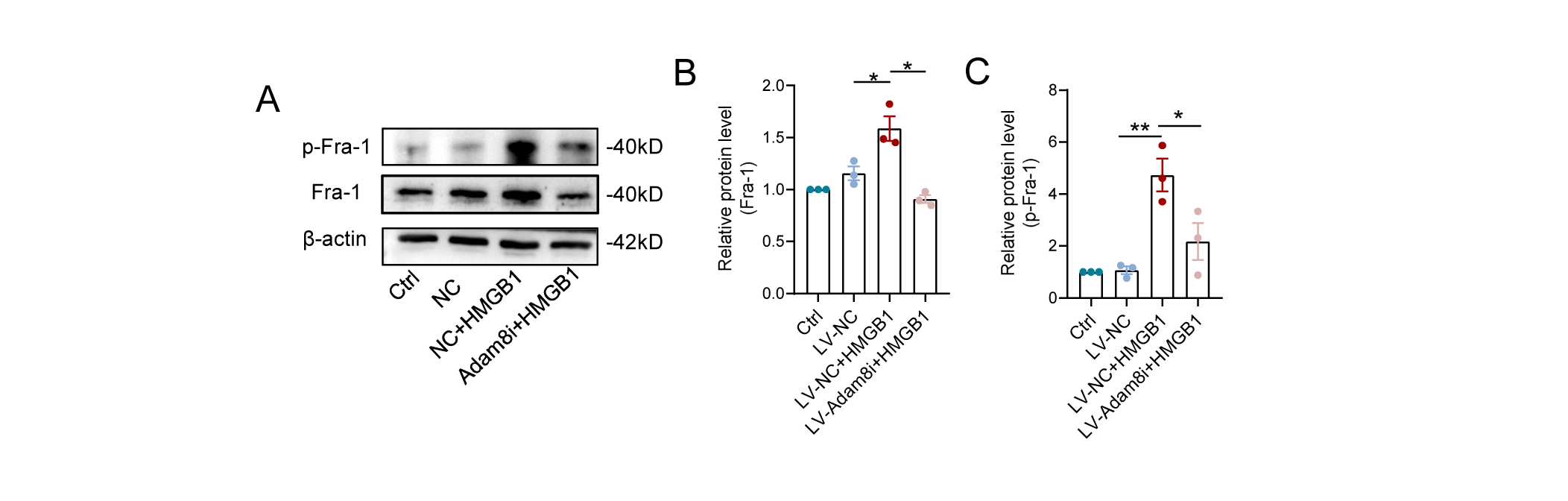

Supplement: Supplementary file 4 — Supplementary Material 4: Fig. S4. Knockdown ADAM8 reduce HMGB1-induced the total and phosphorylated levels of Fra-1 protein in microglial. A Western blot analysis was performed to evaluate the total and phosphorylated levels of Fra-1 protein in microglia treated with HMGB1 and subjected to ADAM8 knockdown or control conditions. β-acin was used as the control. B-C Quantitative analysis of the total and phosphorylated levels of Fra-1 protein. (*p < 0.05, **p < 0.01, and ***p < 0.001). [file 11658_2024_589_MOESM4_ESM.tif]

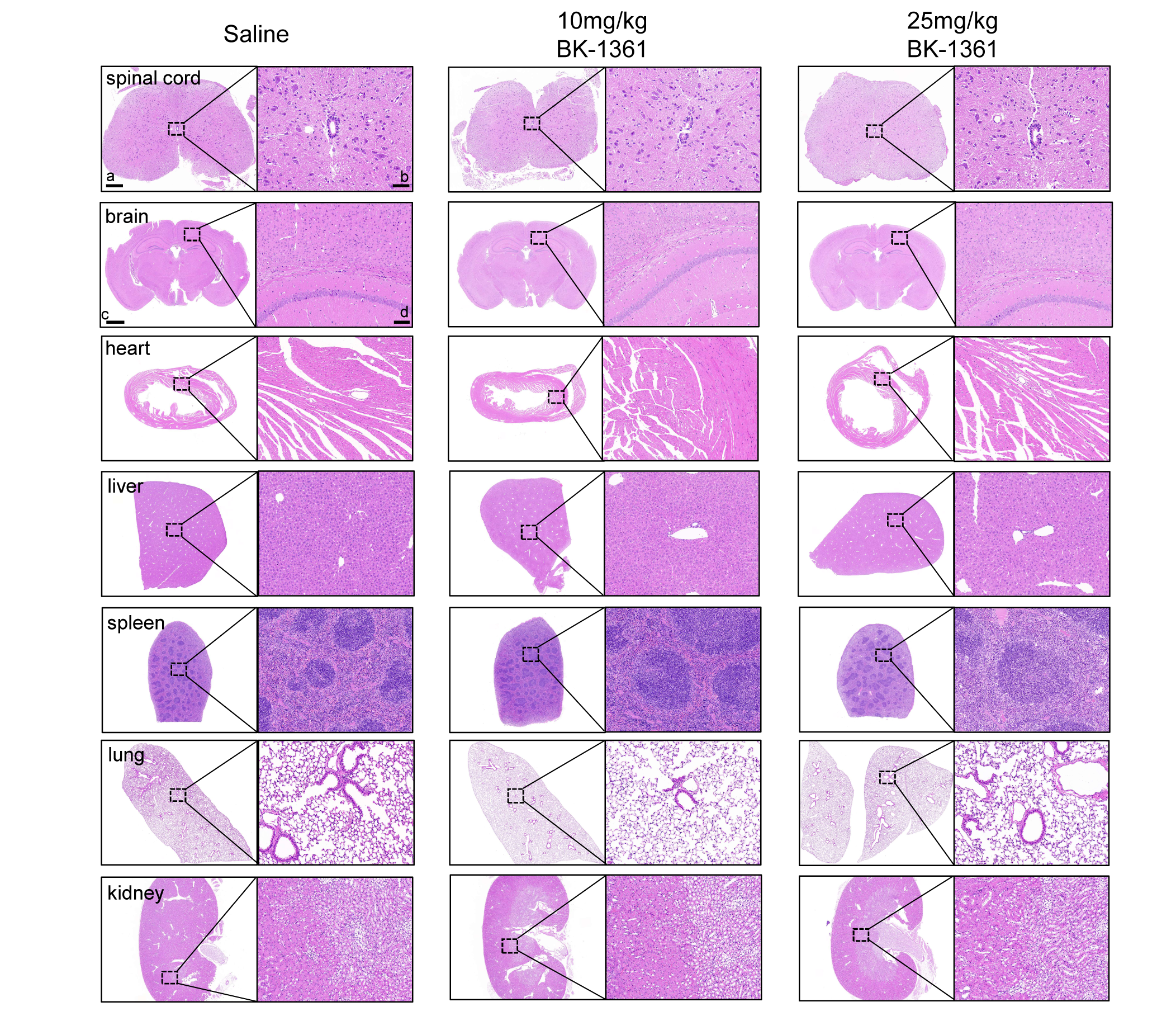

Supplement: Supplementary file 5 — Supplementary Material 5: Fig. S5. HE staining revealed no apparent damage in the mouse spinal cord, brain, heart, liver, spleen, lung, or kidney following treatment with low and high doses of BK-1361. Scale bar: a = 200 μm, b = 50 μm, c = 1 mm, d = 100 μm Fig. S6 Quantitative analysis of the infiltrating cell counting at 3(A) and 7(B) dpi of Fig. 8A. [file 11658_2024_589_MOESM5_ESM.tif]

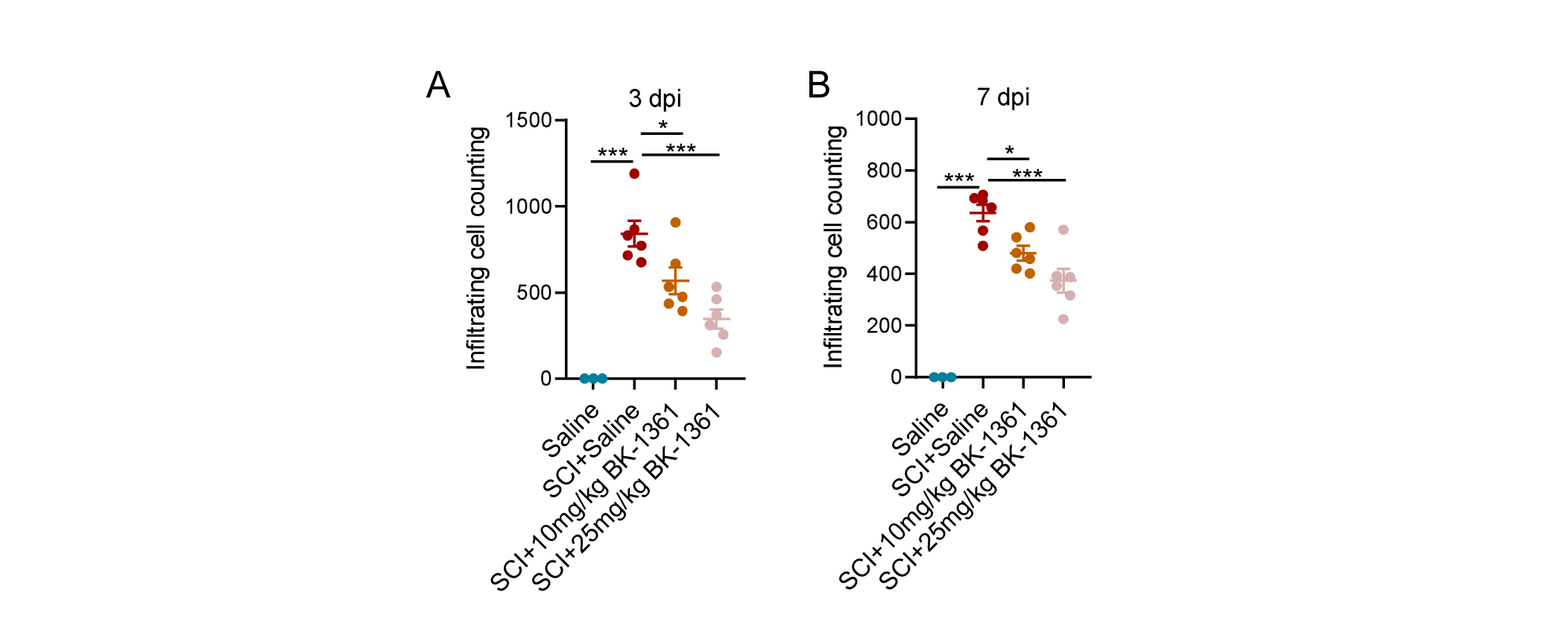

Supplement: Supplementary file 6 — Supplementary Material 6: Fig. S6. Quantitative analysis of the infiltrating cell counting at 3(A) and 7(B) dpi of Fig. 8A. [file 11658_2024_589_MOESM6_ESM.tif]
