# Supplementary material for: Blockade of the ADAM8-Fra-1 complex attenuates neuroinflammation by suppressing the Map3k4/MAPKs axis after spinal cord injury
Source: Cell Mol Biol Lett. 2024 May 16;29:75. doi: 10.1186/s11658-024-00589-3 (PMC11100242; doi:10.1186/s11658-024-00589-3)
Supplement: Supplementary file 7 — Supplementary Material 7: Table.S1. Baseline Characteristics of the Patients. [file 11658_2024_589_MOESM7_ESM.docx]

**Baseline Characteristics of the Patients**

|  | Ctrl | SCI | The value of *P* |
| --- | --- | --- | --- |
| Age, yr (range) | 56.6±1.9 | 59.2±3.8 | *P*=0.5077 |
| Male sex, n | 6 | 9 | *P*=0.4003 |
| Female sex, n | 6 | 3 |  |
| BMI (kg/m^2^) | 24.5±0.8 | 23.6±0.7 | *P*=0.3462 |
| History of diabetes, n | 0 | 0 | *P* >0.9999 |
| History of hypertension, n | 3 | 3 | *P* >0.9999 |
| Current smoker, n | 3 | 2 | *P* >0.9999 |
| Systolic blood pressure, mmHg | 133.6±5.8 | 143.5±5.9 | *P*=0.2434 |
| Diastolic blood pressure, mmHg | 83.2±3.5 | 82.7±3.4 | *P*=0.9191 |

| Name | Segment | Time (h) | Cause |
| --- | --- | --- | --- |
| Lanfang Ding | T9/10 | 45 | vehicle accident |
| Yong Chen | C5/6 | 10 | vehicle accident |
| Yonggen Chen | C3/4 | 20 | fall |
| Dongsheng Zhu | C5/6 | 24 | vehicle accident |
| Rulong Zhao | C3/4 | 35 | vehicle accident |
| Guicai Ju | C4/5 | 6 | fall |
| Wenlan Zhou | C4/5 | 48 | fall |
| Guiying Qian | C3/4 | 30 | fall |
| Rongqing Feng | C4/5 | 37 | fall |
| Binggen Lu | T8/9 | 17 | vehicle accident |
| Cheng Yang | C6/7 | 5 | vehicle accident |
| Chaoyin Sun | C6/7 | 20 | fall |
